# Different leukocyte subsets are targeted by systemic and locoregional administration despite conserved nanomaterial characteristics optimal for lymph node delivery†

**DOI:** 10.1039/d4bm00910j

**Published:** 2024-09-18

**Authors:** Paul A. Archer, Alexander J. Heiler, Alisyn R. Bourque, Yunus Alapan, Susan N. Thomas

**Affiliations:** a Parker H. Petit Institute for Bioengineering and Bioscience, Georgia Institute of Technology IBB 2310 315 Ferst Drive NW Atlanta GA 30332 USA susan.thomas@gatech.edu +1 404-385-1126; b School of Chemical & Biomolecular Engineering, Georgia Institute of Technology Atlanta GA 30332 USA; c George W. Woodruff School of Mechanical Engineering, Georgia Institute of Technology Atlanta GA 30332 USA; d Wallace H. Coulter Department of Biomedical Engineering, Georgia Institute of Technology and Emory University Atlanta GA 30332 USA; e Winship Cancer Institute, Emory University Atlanta GA 30322 USA

## Abstract

Lymph nodes (LNs) house a large proportion of the body's leukocytes. Accordingly, engineered nanomaterials are increasingly developed to direct therapeutics to LNs to enhance their efficacy. Yet while lymphatic delivery of nanomaterials to LNs upon locoregional injection has been extensively evaluated, nanomaterial delivery to LN-localized leukocytes after intravenous administration has not been systematically explored nor benchmarked. In this work, a panel of inert, fluorescent nanoscale tracers and drug delivery vehicles were utilized to interrogate intravenous *versus* locoregionally administered nanomaterial access to LNs and leukocyte subsets therein. Hydrodynamic size and material effects on LN accumulation extents were similar between intravenous *versus* intradermal injection routes. Nanomaterial distribution to various LN leukocyte subsets differed substantially with injection route, however, in a manner not proportional to total LN accumulation. While intravenously administered nanomaterials accumulated in LNs lowly compared to systemic tissues, in sharp contrast to locoregional delivery, they exhibited size-dependent but material-independent access to immune cells within the LN parenchyma, which are not easily accessed with locoregional delivery.

## Abstract 2

Nanomaterial characteristics modulate extent of nanomaterial delivery to lymph nodes but quality of leukocytes accessed varies by administration route.

## Graphical abstract

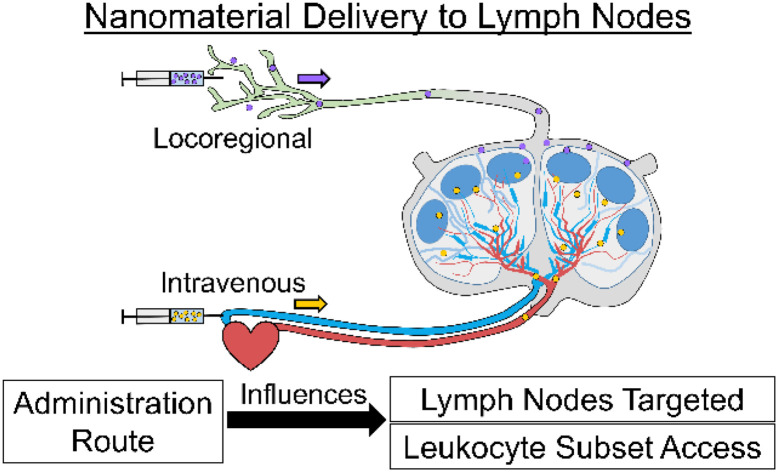

## 1. Introduction

Lymph nodes (LNs) are sites where adaptive immune responses are primed, making them valuable targets for delivery of therapeutics that act upon the immune system.^1,2^ The influence of the immune system is far reaching across numerous categories of diseases and therapeutic interventions including vaccination,^3^ induction of immune tolerance,^4,5^ and cancer immunotherapy,^6^ making LN delivery of interest in a variety of applications.

Central to each of these immune outcomes are interactions between subsets of leukocytes within the LN that play wide-ranging and distinct roles.^7^ Therefore, approaches to enhance the delivery to LNs but also to access particular leukocyte subsets within them are of value to increase control over, and potentially improve, therapeutic effects and outcomes.^8^ However, the LN is a structurally complex organ containing biological barriers that restrict free biomolecular and cellular transport through its structure.^9^ The need for better understanding and control over therapeutic access to cells within the LN motivates the investigation of novel approaches to LN drug delivery.

Nanomaterials often exhibit superior pharmacokinetics compared to molecular therapeutics and are thus widely explored for their drug delivery potential.^10^ This is because systemically administered (*e.g.*, intravenous [i.v.]) nanomaterials exhibit prolonged circulation times^11–15^ and thus can improve LN drug bioavailability.^16^ Nanomaterials administered locoregionally (*e.g.*, intradermal [i.d.], subcutaneous, or intramuscular) also generally exhibit improved profiles of accumulation in LNs draining the injection site by virtue of lymphatic transport.^17–23^ While locoregional administration offers the advantage of targeting specific LNs within distinct lymphatic drainage basins, delivery to systemically distributed LNs can be advantageous for broad drugging of immune cells throughout the body. Because of the complex architecture of the LN, it is difficult to access, and therefore drug, immune cell subsets within the LN parenchyma using conventional delivery approaches. Therefore, there is a pressing need to characterize the nanomaterial influences on LN parenchymal access for various administration routes. I.v. injection is also the standard method of administration for many clinical immunotherapies, and many immunotherapeutic formulations are currently being developed for systemic administration. While careful consideration is given to design therapies with high efficacy and limited adverse side effects due to dissemination into off-target systemic tissues, therapies could potentially be made more effective and safe using nanomaterial formulations.^24^ Furthermore, nanomaterial characteristic effects on access to LN compartments from the lymphatic point of entry have been studied, but direct benchmarking and comparisons of i.v. to locoregional delivery with respect to delivery extents and leukocyte subsets targeted, to inform how different leukocyte subsets may be best targeted (*e.g.* administration route, nanomaterial characteristics), has not been performed.

In this work, fluorescent nanoscale tracers spanning two orders of magnitude in hydrodynamic size were administered i.v. and i.d. to investigate administration route influences on accumulation and cellular association within the LN. These tracers consisted of polymers either widely used to assess *in vivo* lymphatic transport extents and mechanisms^25,26^ or custom synthesized and used in a variety of preclinical drug delivery and immunomodulatory applications.^21,22,27–37^ Comparing the overall pattern of LN accumulation amongst the nanomaterial panel, characteristics regulating LN accumulation extent were consistent between administration routes. However, the subsets of leukocytes associated with administered nanomaterials varied substantially, with i.v. delivery enhancing association with LN parenchyma resident cells, including lymphocytes. When considering size and material influences on LN delivery from i.v. administration, association with LN-localized leukocytes was nanomaterial hydrodynamic size-dependent but not directly proportional to the total quantity of nanomaterial accumulating within the tissue. When accounting for the total level of LN accumulation, the extent of association with various LN leukocyte populations was material-independent, implicating size as a dominant influence on access to cells within the LN from i.v. administration. Thus, i.v. administered nanomaterial delivery to the LN is size-dependent both in terms of total delivery and access to cells, and enhances direct access to LN parenchyma-resident leukocytes compared to i.d. administration. These results lay the foundation for the design of nanoscale biomaterial approaches to achieve LN delivery and access to specific leukocyte subsets to improve systemic immunotherapy outcomes.

## 2. Materials and methods

### 2.1. Fluorescent tracers

Tetramethylrhodamine isothiocyanate (TRITC) 2 MDa dextran, Texas Red 70 kDa dextran, Alexa Fluor 647 10 kDa dextran, and TRITC 10 kDa dextran (TRITC excitation/emission spectrum 555/575 nm and Texas Red excitation/emission spectrum 595/615 nm) were purchased from Thermo Fisher Scientific. Fluorophores were surface conjugated to these dextran tracers by the manufacturer through succinimidyl coupling to yield a stable amide bond. Carboxylate-modified 0.5 μm red, 0.05 μm orange, 0.05 μm dark red, 0.02 μm yellow-green, and 0.02 μm crimson polystyrene microspheres (Fluospheres®, excitation/emission spectra 580/605, 540/560, 625/645, 660/680, and 505/515 nm, respectively) were purchased from Thermo Fisher Scientific. These tracers were dye-labeled by the manufacturer in a proprietary procedure of swelling the polystyrene microsphere in a solvent containing hydrophobic dye to allow the dye to diffuse into the polystyrene particle, then reversing the swelling in an aqueous solvent to trap dyes within the particle. Five-hundred kDa and 40 kDa amino dextran (Thermo Fisher Scientific) were labeled by incubation with Alexa Fluor 700 (AF700, excitation/emission spectrum 700/720 nm) and Alexa Fluor 647 (AF647, excitation/emission spectrum 645/665 nm) NHS (*N*-hydroxysuccinimide) ester dyes in 0.1 M NaHCO_3_ at pH 8.1 overnight on an orbital rotator, yielding a stable amide bond through succinimidyl coupling. Dextran-fluorophore conjugates were purified from unreacted dye using a Sepharose CL-6B size exclusion column (Sigma Aldrich) and purified dextran tracers were concentrated by centrifugal spin filter (Millipore Sigma) before use. Final concentrations of dextran tracer solutions were quantified by phenol-sulfuric acid colorimetric carbohydrate detection assay by comparison to a glucose standard curve (Cell BioLabs). All tracers were maintained in sterile conditions.

### 2.2. NP synthesis

Poly(propylene sulfide) NPs (PPS NPs) were synthesized as described previously^22,36–38^ with procedural modifications to yield final NP products of three different sizes. Briefly, to synthesize 30, 80, and 500 nm PPS NPs, respectively, either 500, 25, or 2.5 mg of Pluronic F127 (Sigma Aldrich) was dissolved in 10 mL degassed Milli-Q water for 30 minutes with stirring. Subsequently, 400 μL (5 mmol) of propylene sulfide (Sigma Aldrich) was added under argon for 30 minutes, while stirring. Initiator (S,S′-(2,2-bis((acetylthio) methyl)propane-1,3-diyl)diethanethioate) (0.04 mmol) was activated with 322 μL sodium methoxide (Sigma Aldrich) for 15 minutes, and then added to the reaction mixture under argon. 1,8-Diazabicyclo[5.4.0]undec-7-ene (0.4 mmol) was added to the reaction under argon and reacted for 24 h. Cross-linking of the NP core was facilitated by exposing the reaction mixture to air for 2 h with stirring, and the reaction mixture was then dialyzed for three days using 100 kDa MWCO cellulose membrane dialysis tubing (SpectrumLabs). After dialysis, 30 nm and 80 nm NPs were pushed through an Acrodisc 0.2 μm sterile filter (Pall Corporation) and all NPs were maintained in sterile conditions. Concentration of free thiols in the NPs was measured by Ellman's assay (Thermo Fisher Scientific) and NP size was measured by dynamic light scattering on a Zetasizer Nano ZS (Malvern Panalytical). Core thiols were capped using either Alexa Fluor 647, Alexa Fluor 488, or Texas Red maleimide for 4 h, followed by excess *N*-ethylmaleimide (to a final concentration of 32 mM) in 1× PBS overnight to cap any remaining core thiols. Unreacted dye was cleaned from dye-labeled PPS NPs either by dialysis for three days using 100 kDa MWCO cellulose membrane dialysis tubing (SpectrumLabs) or by Sepharose CL-6B (Sigma Aldrich) size exclusion chromatography column. Prior to injection into animals, NPs were concentrated using 30 kDa MWCO Amicon Ultra centrifugal filter (Millipore) at 4000*g*.

### 2.3. Fluorescent tracer and PPS NP injections

Tracers and PPS NPs were administered in a 20 μL bolus injection for i.d. administration and 200 μL bolus injection for i.v. administration. Each tracer size was labeled with a fluorescent dye (Fig. S1A†) and fluorescent tracers were co-administered in combinations designed with minimal spectral overlap. Spectral overlap was accounted for through fluorescence compensation, such that accumulation of each tracer could be quantified independently of other co-injected components. For tissue homogenate experiments, injected amounts of material were selected to ensure the quantity reaching the LN provided a fluorescent signal above tissue background (Fig. S1B†) and that the limit of detection was within an order of magnitude of all others (Fig. S1C†), such that each fluorescent probe was injected in an amount that would facilitate consistent detectability that was comparable to that of all others.

Similarly, for flow cytometry experiments, administered quantities of each tracer were selected to maintain the limit of detection of tracer positive signal within cells within a threshold similar to all others such that signal from all tracers would be detectable and comparable (Fig. S2A†), which was assessed by incubating a constant quantity of murine splenocytes with serial dilutions of tracer until tracer signal within cells was no longer distinguishable from background when analyzed by flow cytometry on the basis of median fluorescence intensity (MFI) (Fig. S2B†). While injected quantities of all tracers were safely above the lower detection limit, the degree of labeling of the 500 nm polystyrene tracer was such that it was detectable on a single cell basis at lower concentrations than the other tracers (Fig. S2C†).

Tracers with similar fluorophore labels, for which fluorescence compensation would be insufficient, were administered to separate animal cohorts. All PPS NP sizes were injected individually without co-injection. Mice were maintained under anesthesia *via* isoflurane throughout the tracer injection procedure, following all institutional guidelines and approvals of the Institutional Animal Care and Use Committee.

### 2.4. Quantification of total fluorescence within tissue homogenates from *in vivo* biodistribution studies

C57Bl6 mice were purchased from Jackson Laboratories at 6–8 weeks of age. All animal procedures were approved by and adhered to guidelines set forth by the Institutional Animal Care and Use Committee of the Georgia Institute of Technology. In tissue homogenate biodistribution experiments in healthy animals, 6–8 weeks old mice were injected i.v. or i.d. with tracer solution, and at 1, 4, 24, and 72 h post tracer injection, cohorts of animals were anesthetized for blood collection *via* cardiac puncture, then animals were sacrificed for tissue dissection. Axillary, brachial, and inguinal lymph nodes were collected from both sides of the mouse, as well as the spleen, kidneys, liver, lungs, and blood. In the case of i.d. injections, the skin at the injection site was also excised. Each tissue was weighed and homogenized in a homogenization tube loaded with 1.4 mm acid-washed zirconium beads (OPS Diagnostics) and 400 μL PBS using a FastPrep-24 automated homogenizer (MP Biomedical). Homogenized tissue solutions were then transferred to a 96-well plate to measure the fluorescence in each tissue using a Synergy H4 BioTek plate reader. Fluorescence compensation was applied to all samples from animals receiving co-injected tracer. Fluorescence standard curves for each fluorescently labelled nanomaterial were performed by adding increasing known amounts of the fluorescent nanomaterial to homogenized tissue and reading the values on a BioTek plate reader. Sample fluorescent signal from each tracer type was then converted to quantity of tracer present in the tissue using these fluorescence standard curves in tissue homogenate. Tissues were weighed after removal from the animal and total tissue mass was used to scale up tracer present in measured quantities of tissue homogenate to tracer present within the entire organ. For results presented as percent injected dose per tissue volume, tissue masses were converted to volumes approximating the organ density to that of water.

### 2.5. Flow cytometry analysis from *in vivo* biodistribution studies

For cell association biodistribution experiments, mice were injected i.v. or i.d. with tracer solution and sacrificed 4 hours post tracer injection. Left and right side axillary, brachial, and inguinal lymph nodes, and spleen from each mouse were collected and processed as described in the following section. Lymph nodes were incubated in collagenase D (1 mg mL^−1^) (Sigma Aldrich) in PBS for 1 hour at 37 °C, and then all tissues were processed into single cell suspension by gently pushing through a 70 μm cell strainer (Greiner Bio-One) with a 1 mL syringe plunger and washing through with PBS. Single cell suspensions from spleen samples were incubated with red blood cell lysis buffer (Sigma Aldrich) for 7 minutes, diluted with PBS, washed, and re-suspended. Single cell suspensions from each tissue were centrifuged at 400*g*, 5 min, 4 °C, decanted, re-suspended, and transferred to a round bottom 96 well plate for staining. All cell staining antibodies were purchased from BioLegend. Samples were incubated with 2.5 μg mL^−1^ CD16/CD32 (2.4G2) Fc block (Tonbo Biosciences) for 5 min on ice, washed, and then incubated with Zombie Aqua viability stain for 30 min at room temperature and washed. Cells were incubated with a solution of surface staining antibodies suspended in FACS buffer (0.1% bovine serum albumin in PBS) for 30 min on ice. In experiments with tracer injections of TRITC 10 kDa dextran, AF700 500 kDa dextran, and yellow-green 0.02 μm polystyrene particle, cells were stained with BV711 anti-mouse CD3, BV605 anti-mouse CD169, APC/Cy7 anti-mouse CD45, BV650 anti-mouse B220, BV421 anti-mouse CD11c, BV785 anti-mouse F4/80, and PE/Cy7 anti-mouse CD11b. In experiments with tracer injections of 0.5 μm red polystyrene particle, 0.05 μm dark red polystyrene particle, 0.02 μm yellow-green polystyrene particle, and AF700 500 kDa dextran, cells were stained with APC/Cy7 anti-mouse CD3, PE/Cy7 anti-mouse CD169, BV650 anti-mouse B220, BV711 anti-mouse CD11c, BV785 anti-mouse F4/80, and BV421 anti-mouse CD11b. In experiments with tracer injections of AF647 40 kDa dextran, Texas Red 70 kDa dextran, AF700 500 kDa dextran, and TRITC 2 MDa dextran, cells were stained with APC/Cy7 anti-mouse CD3, PE/Cy7 anti-mouse CD169, BV650 anti-mouse B220, FITC anti-mouse CD11c, BV785 anti-mouse F4/80, and BV421 anti-mouse CD11b. Following antibody staining, cells were washed, then fixed by incubation in 2% paraformaldehyde solution for 15 min on ice, before final wash in FACS buffer and storage at 4 °C. Samples were analyzed by flow cytometry on a BD LSRFortessa.

### 2.6. Confocal microscopy

For LN imaging studies, Texas Red labeled 30 nm PPS NPs were i.d. administered in the hind limb footpad (lymphatic draining to the inguinal LN) and Alexa Fluor 647 labeled 30 nm PPS NPs were i.v. administered in the jugular vein. Animals were sacrificed 4 h post-administration and inguinal LNs were excised, placed in cryomolds, and submerged in OCT embedding medium. Cryomolds were placed under vacuum at 0.8 bar for 5 minutes, then placed in a beaker containing isopentane below the height of the cryomolds, which was then placed in liquid nitrogen to freeze the samples. Once frozen, cryomolds were transferred to −80 °C storage. LNs were sectioned on a CryoStar NX70 Cryostat (Thermo Scientific) at 10 μm thickness, mounted on slides, and fixed. Images were captured on a Zeiss 900 laser scanning confocal microscope at 20× magnification.

### 2.7. Statistical analysis

All data are expressed as mean ± SEM unless otherwise noted. Statistical analyses were performed in GraphPad Prism 9. Comparisons involving three or more groups are made by one-way ANOVA with Tukey's multiple comparison test, or by Welch ANOVA with Dunnett's T3 multiple comparison test if Brown–Forsythe test for differences between standard deviations within groups is significant. Two-way ANOVA is used in comparisons in which time is considered as an additional variable affecting the dependent variable outcome. Kendall's Tau coefficient tests were performed in R Studio version 3.6.3 using the cor. test function, method = “Kendall”. For statistical comparisons, *P* values are denoted as follows: * = *p* < 0.05, ** = *p* < 0.01, *** = *p* < 0.001, **** = *p* < 0.0001, or alternatively, # = *p* < 0.05, ## = *p* < 0.01, ### = *p* < 0.001, #### = *p* < 0.0001. Significance was considered *p* < 0.05.

## 3. Results

### 3.1. Nanomaterial delivery to the LN is size and temporally dependent, in a manner consistent across systemic and locoregional administration methods

**Fig. 1:**
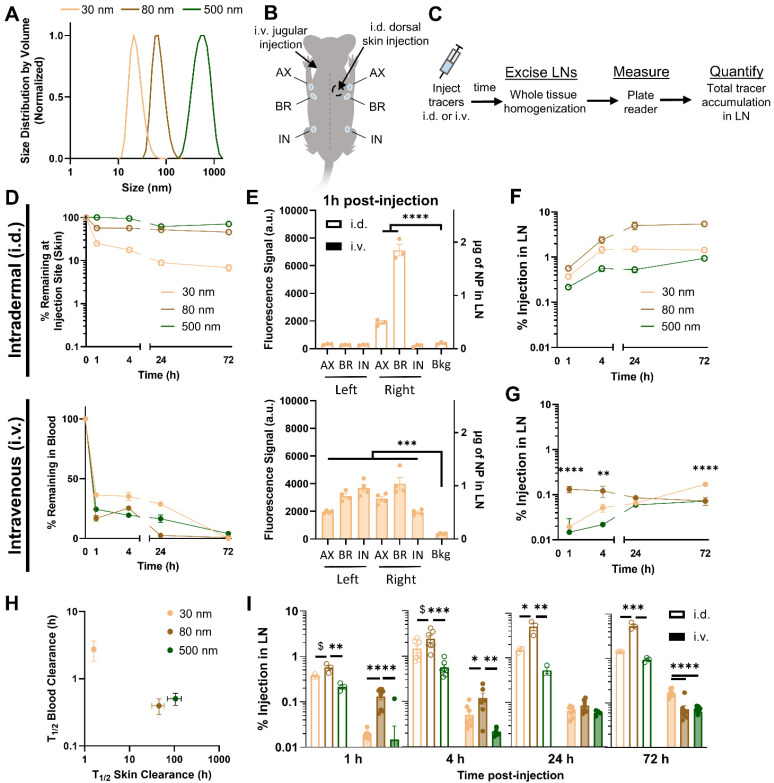
Hydrodynamic size effects on LN accumulation from i.d. and i.v. administration for PPS NPs. (A) Hydrodynamic size of PPS NP measured by dynamic light scattering. (B) Schematic indicating anatomical locations of LNs analyzed in biodistribution studies. Location of i.d. administration was in the superior right dorsal skin. (C) Experimental workflow for quantifying NP tissue accumulation. (D) Percentage of NP dose remaining at the skin injection site post i.d. injection (top) and within the blood post i.v. injection (bottom). (E) Measured fluorescent signal and associated mass of NP, as calculated from fluorescence standard curves in tissue homogenate, detected in each of the axillary (AX), brachial (BR), and inguinal (IN) LNs 1 h post i.d. (top) and i.v. (bottom) administration. *** and **** (*p* < 0.001 and *p* < 0.0001, respectively) indicate significant difference from background (Bkg) fluorescent signal by one-way ANOVA. Time resolved accumulation of PPS NP in the right brachial LN determined from endpoint tissue homogenate measurements from i.d. (F) and i.v. (G) administration. ** and **** indicate significant difference in LN accumulation of designated NP size from other NP sizes at specified time point of analysis. (H) Half life of clearance from blood after i.v. administration and clearance from skin after i.d. administration, as determined from one phase decay fit from (D). (I) Comparison of size trends in LN accumulation from i.d. and i.v. administration at 1, 4, 24, and 72 h post NP administration. *, **, ***, **** indicate significant difference between groups (*p* < 0.05, 0.01. 0.001, 0.0001, respectively) by one-way ANOVA. $ indicates significant difference between groups at the threshold *p* < 0.1. *n* = 3–7 mice per group.

To investigate the size-dependence of LN accumulation for i.d. and i.v. administered nanomaterials, fluorescent poly(propylene sulfide) (PPS) NPs were administered in a bolus injection into the lateral dorsal skin or jugular vein, respectively. I.d. administered NPs were injected at the same concentrations used in i.v. injection (Fig. S1A†), but at a tenfold lower volume, which was both a necessity in order to maintain the fluorescence signal from the two administration routes above limit of detection (Fig. S3†) and a clinically appropriate approach, given that locoregional administration methods are typically performed with lower injection volumes.^39^ The PPS NPs used in these studies that have been used for various therapeutic lymphatic drug delivery applications by our lab and others^21,22,28,34,37^ were synthesized at 30, 80, and 500 nm in size (Fig. 1A and Fig. S4†), modifying the protocol of Rehor *et al.*^40^ Therefore, this NP system was applied in these studies without drug loading to evaluate size-dependent biodistribution of a relevant nanomaterial drug delivery system. Following NP administration, their LN accumulation was assessed in each of the axillary, brachial, and inguinal LNs (Fig. 1B) by endpoint biodistribution analysis. Cohorts of animals were sacrificed at 1, 4, 24, or 72 h post-injection, at which point tissues were excised and fluorescent NP signal in bulk tissue homogenate of each tissue was quantified (Fig. 1C).

I.d. administered NPs were cleared from the dorsal skin injection site in a manner inversely related to NP size; while the 500 nm NP were mostly retained in the skin at all time points tested, the majority of the 30 nm NP was cleared within just 1 h (Fig. 1D, top). By contrast, when administered i.v., the 30 nm NP was cleared slowly, with approximately 30% of injected dose remaining in circulation after 24 h, whereas the 80 and 500 nm sizes were cleared more quickly (Fig. 1D, bottom).

As expected, administration route substantially influenced NP distribution to LNs in different anatomical locations. Whereas i.d. administered NPs were detectable within only the ipsilateral, axillary, and brachial LNs in the local lymphatic drainage basin, i.v. administered NPs were detectable in all evaluated LNs (Fig. 1E), draining both peripheral and deep organ tissues (Fig. S5†). Considering the brachial LN is the primary draining LN from this dorsal skin injection site, and NP delivery to all LNs was similar from i.v. administration (Fig. 1E), subsequent comparisons between administration routes are made on the basis of accumulation within the brachial LN, for simplicity.

NP accumulation in the draining LNs upon i.d. injection was hydrodynamic size-dependent, with accumulation of the 80 nm NP greater than that of the 30 nm NP, and both superior to the 500 nm NP across all time points from 1 h to 72 h (Fig. 1F). This trend was also observed when NPs were administered i.v. 1 and 4 h post-injection, but diminished at later time points as the 30 nm NP consistently increased in LN accumulation and became the dominant size accumulated in the LN by 72 h (Fig. 1G). This likely resulted from the characteristic differences in blood half-life of each NP size (Fig. 1H), resulting in more sustained circulation and LN accumulation of the 30 nm NP, while accumulation of the 80 nm NP instead diminished with time (Fig. 1I). While the time-dependent factors of blood and tissue retention become increasingly influential on endpoint readouts at later time points of analysis, data up to 4 h post-administration, when NPs of all sizes are still retained at considerable amounts, indicated a consistent size trend in LN accumulation for both i.d. and i.v. delivery. Therefore, tracer delivery was analyzed 4 h post-injection to limit cell-mediated transport/recirculation effects and the influence of differential LN retention and blood circulation times between materials,^23,41^ and because i.v. and i.d. PPS NP tracers were at a similar point in their LN accumulation profile at this time point.

**Fig. 2:**
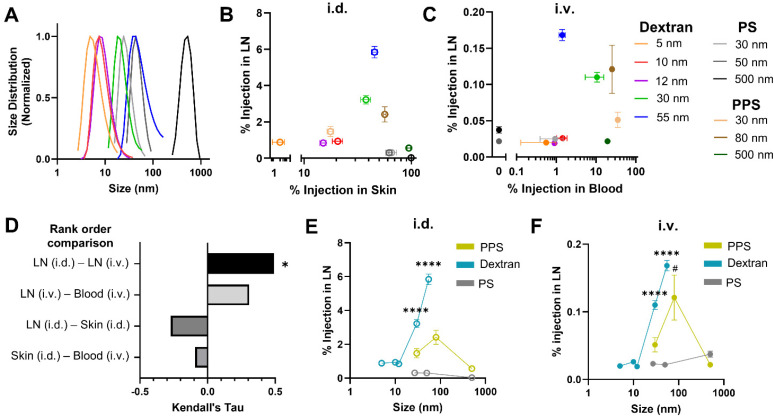
Efficiency of LN accumulation amongst PPS NPs and macromolecule dextran and polystyrene particle tracers is similar between i.d. and i.v. administration routes and comparably regulated by nanomaterial hydrodynamic size. (A) Hydrodynamic size of dextran and polystyrene tracers measured by dynamic light scattering. (B) Percent injected tracer amount in LN *versus* within the skin injection site 4 h post i.d. administration. (C) Percent injected tracer amount in LN *versus* blood 4 h post i.v. administration. (D) Correlation between ranked order of tracer quantities measured within the LN, skin, and blood from i.d. and i.v. administration, quantified by Kendall's Tau. * indicates significant correlation between tracers most effective for i.d. and i.v. LN accumulation (*p* < 0.05). Size dependence of LN accumulation for PPS, dextran, and PS tracers 4 h post i.d. (E) and i.v. (F) administration. **** indicates significant difference (*p* < 0.0001) between designated dextran tracer size and all other dextran tracer sizes, whereas # indicates significant (*p* < 0.05) difference between designated PPS NP size and all other PPS NP sizes by one-way ANOVA.

To probe the generalizability of this finding, additional studies were performed with a panel of dextran and polystyrene tracers that are widely used for studying lymphatic transport and locoregional administration effects on biodistribution^20,21,27,41–44^ because they are inert and are available in a biologically relevant range of sizes (Fig. 2A). Tracer compositions were selected to span a range of hydrodynamic sizes that both overlap with the PPS NPs already tested and are well-documented to exhibit prodigious lymphatic uptake and LN accumulation after locoregional administration. To reduce the biological variability between animals in these studies as well as enable intra-cohort comparisons, co-injection of tracers of different sizes with unique fluorophore labels (Fig. S1†) was utilized. Co-injection of dextran and polystyrene tracer materials into the same animal was confirmed not to alter tracer biodistribution to systemic organs (Fig. S6A†) or LNs (Fig. S6B†) as compared to individual injections.^20^ Tracer solutions were administered either i.d. or i.v. and animals were sacrificed for endpoint tissue fluorescence analysis 4 h post tracer administration.

When total tracer quantities accumulating within the LN were compared from tissue homogenate studies, a significantly higher percentage of the i.d. dose of tracers accumulated in the brachial LN (here, the principle LN draining the skin injection site) than accumulated from i.v. administration, with the exception of the 500 nm polystyrene tracer that was not detectable in the brachial LN when i.d. injected, presumably due to its size and rigidity inhibited substantial passive lymphatic drainage within the 4 h timescale tested^41^ (Fig. S7A†). This result was not surprising given the inherent differences in selectivity for LN accumulation of locoregional as opposed to systemic methods of administration. In fact, even when compared on the basis of raw mass of tracer reaching the LN, despite injecting a greater volume of tracer i.v., i.d. administration still resulted in greater LN accumulation for all dextran tracers as well as the 30 and 80 nm PPS NP (Fig. S7B†).

For i.d. administered tracers, while materials very lowly and highly retained in the skin injection site accumulated in the LN less effectively, maximum LN accumulation occurred for the 30 nm dextran, 55 nm dextran, and 80 nm PPS NP, which remained approximately 40–55% retained within the skin at 4 h (Fig. 2B). Thus, LN accumulation from i.d. administration peaked for materials displaying sufficient retention within the skin to bias delivery toward lymphatic vasculature by interstitial flow, but not so much retention that clearance from the skin was stifled. When administered i.v., the 5, 10, and 12 nm dextran tracers and all polystyrene tracers were cleared from the blood within 4 h and displayed limited LN accumulation (Fig. S8† and Fig. 2C). LN accumulation was elevated for dextran and PPS materials 30–80 nm in size with generally greater retention in the blood, though for those tracers with increasing levels remaining in blood circulation, LN accumulation diminished (Fig. 2C). This suggested LN accumulation from i.v. administration necessitated circulation time within the blood above a certain threshold, but for very long circulating materials, such as the 30 nm PPS NP, this also delayed LN accumulation until later time points (Fig. 1I). Correlation between the tracers most effective for LN accumulation from i.d. and i.v. administration was quantified by Kendall's Tau coefficient. In this analysis, tracers of all compositions and sizes were ranked on the basis of percentage of injected quantity detected within the LN, and Kendall's Tau coefficient was used to measure similarity between the ranked order of tracers most accumulated within the LN from i.d. *vs.* i.v. administration. Tracers most effective for accumulation within the LN from i.d. and i.v. administration were significantly positively correlated, indicating tracer accumulation profiles within the LN were similar in a manner independent of administration route (Fig. 2D). In contrast, when this analysis was performed on the ranked order of tracers most effective for LN accumulation *vs.* greatest retention in the skin or blood, correlations were not significant (Fig. 2D). Interestingly, from both i.d. and i.v. administration, LN accumulation of all tracers followed a similar size-dependent trend, with dextran and PPS NPs displaying elevated delivery in the 30–80 nm size range (Fig. 2E and F), despite the compositional and physiochemical differences of these materials. Notably, while their relative LN accumulation in relation to the other tracer materials was consistent between both administration routes, the polystyrene tracers did not match the size trend of the dextran and PPS NPs (Fig. 2E and F), perhaps because of their rigidity and hydrophobicity^20^ which are characteristics known to diminish circulation time upon i.v. administration^45,46^ and lymphatic delivery upon i.d. administration.^20^ Indeed, the relatively higher skin retention of the polystyrene tracers when administered i.d. (Fig. 2B) and substantially faster clearance from blood of the polystyrene tracers when administered i.v. (Fig. 2C and Fig. S8†) indicated material properties less favorable for LN delivery. Overall, nanomaterials with size and material characteristics that promoted moderate retention in either the skin or blood after i.d. or i.v. injection, respectively, mainly hydrophilic tracers between 30–80 nm, exhibited elevated accumulation within the LN over a 4 h timeframe post injection.

### 3.2. Size-dependent tracer accumulation profile in LNs differs from accumulation profile in systemic organs

In addition to LN delivery, i.v. administered tracer accumulation within systemic organs was assessed, given the potential implications of off target delivery on therapeutic outcomes.^47,48^ While the systemic biodistribution of i.v. administered tracers is well established,^49,50^ the dependence on tracer size and material and comparison to LN distribution was examined.

Systemic organ exposure of each fluorescent tracer was measured and percent of injected tracer quantity accumulated in the liver, spleen, lungs, and kidneys at each time point of sacrifice calculated (Fig. S9A–D†). In general, total accumulation of tracer in the systemic organs was higher than that in any individual LN, as may be expected given the substantially larger tissue volumes of systemic organs. By adjusting for tissue volume of each organ, the concentration of tracer delivered to each organ were compared. The concentration of 30 nm PPS NP was notably highest in the blood from 1–24 h, highlighting the long circulation time of the PPS NP, likely afforded by PEGylation, which is widely appreciated to prolong nanomaterial circulation times,^51^ while concentrations within the LN and liver remained similar at all times (Fig. S10A†). Concentrations of the 80 nm and 500 nm PPS NP and all polystyrene tracer sizes were significantly higher in the spleen, liver, or lung rather than the LN for all sizes and time points tested (Fig. S10B, C and I–K†). Tracer concentrations within the LN were low across all time points for dextrans of size 5–12 nm, superseded by concentrations in the kidneys or liver (Fig. S10D–F†). By contrast, for the 30 nm dextran, concentration within the LN was initially not significantly different from that in the liver but became significantly higher after 72 h (Fig. S10G†), whereas for the 55 nm dextran, concentration within the LN became significantly higher than all systemic organs from 24–72 h post-injection (Fig. S10H†). Thus, from i.v. administration, as a function of size, concentration of dextran within the LN increased relative to systemic organs in the 30–55 nm size regime, whereas for PPS NP polystyrene particles, concentration within the LN remained at or below concentrations within systemic organs across all sizes tested (Fig. S11†).

**Fig. 3:**
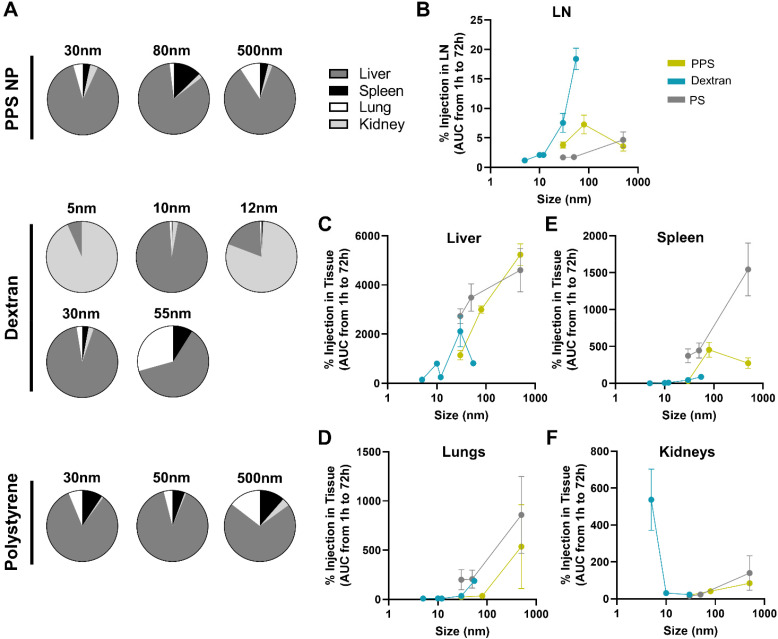
Hydrodynamic size dependency of tracer accumulation over 72 h post i.v. administration diverges for systemic organs *versus* LNs. (A) Relative contribution of the liver, spleen, lungs, and kidneys to the total systemic organ accumulation measured for each tracer. Size dependence of tracer accumulation within the (B) LN, (C) liver, (D) lungs, (E) spleen, and (F) kidneys presented as area under the curve of time resolved accumulation profile presented in Fig. S13.† 12 nm Texas Red labeled and 55 nm TRITC labeled dextran tracer data is excluded from (F) due to high autofluorescence of these fluorophores in kidney tissue homogenate. *n* = 3 to 7 mice at each time point and results are representative of at least two independent experiments.

Overall tracer accumulation within each tissue was compared by calculating area under the curve (AUC) of time-resolved tracer delivered to each tissue from 1 to 72 h (systemic organ data presented in Fig. S9,† LN data presented in Fig. 1G and Fig. S12†). Independent of tracer material, the liver was the dominant systemic organ in which tracer accumulated for all tracer sizes except the 5 and 12 nm dextran, which were detected in greater quantities in the kidneys (Fig. 3A). Tracer accumulation within the liver, spleen, lungs, and kidneys was also size dependent, as has been reported by others,^45^ but notably in a manner differing from that in the LN (Fig. 3B). Tracer accumulation within the liver, lungs, and spleen trended upward with increasing size across all materials (Fig. 3C and D), with the exception of the 500 nm PPS NP that deviated from this trend with less accumulation in the spleen than the 80 nm size (Fig. 3E). In general, polystyrene tracers accumulated to greater extents in the liver and spleen than dextran tracer and PPS NPs of similar size, suggesting a material-driven effect (Fig. 3C and E). By contrast, accumulation in the kidneys peaked at smaller sizes (Fig. 3F). This indicates the size dependence of accumulation within the LN observed for the dextran and PPS NP is unique to that tissue and not just a property of the distribution of these materials across the body at large.

### 3.3. I.v. administration biases nanomaterial delivery to LN-resident lymphocytes and away from barrier macrophages compared to i.d. administration

Given that i.v. administration delivers materials directly into the circulation, we hypothesized that the LN's blood vasculature would be a dominant route of entry into the tissue for tracers injected i.v. This is in contrast to locoregional methods of delivery, such as i.d. administration, that are commonly employed to leverage preferential delivery to the LN through its lymphatic vasculature. Given the size-restrictive nature of the LN subcapsular sinus (SCS) that limits direct lymphatic-mediated access of solutes >70 kDa in molecular weight to the LN parenchyma,^52–54^ the potential for i.v. administration to enhance access of larger nanomaterials to cell types housed within the LN parenchyma was explored.

**Fig. 4:**
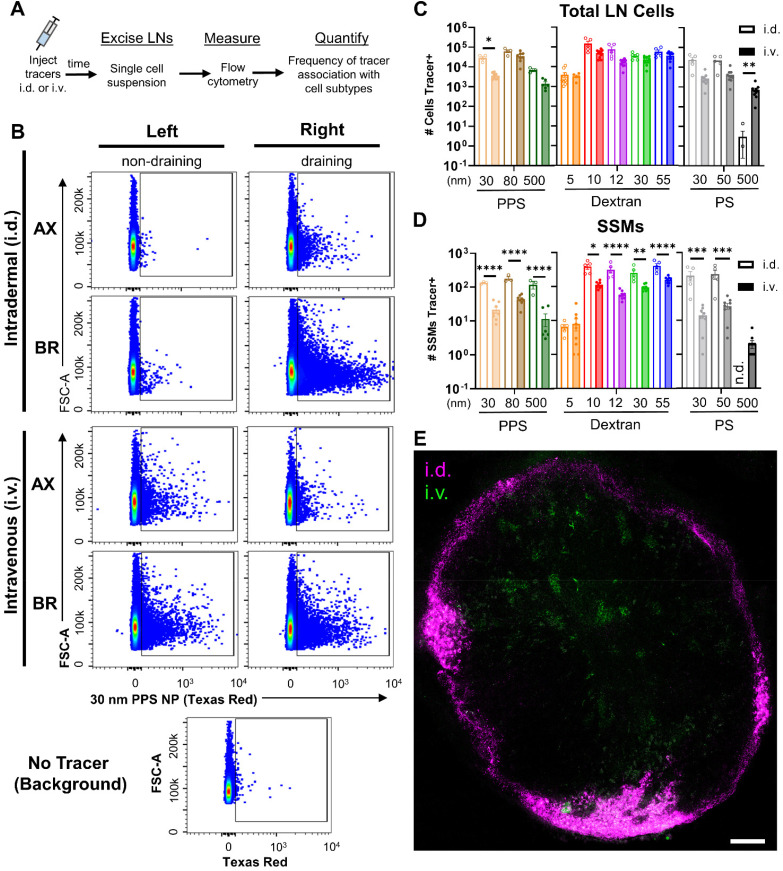
Tracer distribution to LN-localized cell subsets varies between intravenous *versus* intradermal routes of administration. (A) Experimental workflow flow cytometrically quantifying tracer association with leukocyte subsets within LNs. (B) Representative flow cytometry plots of fluorescent tracer associated with leukocytes in axillary (AX) and brachial (BR) LNs collected from mice either i.d. or i.v. administered 30 nm PPS NP. LNs collected from mice not administered tracer were used to define tracer positive signal above background in experimental samples. (C) Number of cells within the LN associated with tracer from i.v. and i.d. administration. *, ** indicate significant difference (*p* < 0.05, *p* < 0.01, respectively) between i.v. and i.d. administration for each tracer size as determined by Welch ANOVA. Data are presented as mean ± SD. (D) Number of SSM (CD3^−^B220^−^CD11b^+^CD169^+^F4/80^−^CD11c^LO^) within the LN associated with tracer from i.v. and i.d. administration. *, **, ***, **** indicate significant difference (*p* < 0.05, *p* < 0.01, *p* < 0.001, *p* < 0.0001, respectively) between i.v. and i.d. administration for each tracer size as determined by Welch ANOVA. Data are presented as mean ± SD. (E) Confocal microscope image of 30 nm PPS NP distribution within the LN 4 h post i.v. (AlexaFluor 647 labeled, green) and i.d. (Texas Red labeled, magenta) administration. Scale bar represents 100 μm. In A–D, *n* = 5 mice per group and results are representative of two independent experiments.

These studies were carried out following the same workflow utilized to assess bulk tissue accumulation of tracers, but rather than measuring fluorescent tracer signal in tissue homogenates, samples were processed into single cell suspension and stained for immune phenotyping *via* flow cytometry (Fig. 4A and Fig. S13†). As was true in total tissue accumulation studies, administration route determined the distribution of LNs to which tracer was delivered to cells. I.d. administered tracers were associated with cells predominantly within the local draining LNs, whereas i.v. administered tracers associated with LN cells throughout the body (Fig. 4B). Gate placement for positive tracer signal associated with cells was determined using uninjected negative control LN samples, which confirmed signal in LNs was above background (Fig. 4B and Fig. S14†).

Total accumulation of tracer within the LN homogenate was enhanced through i.d. delivery. Nonetheless, when delivery of tracers to cells was assessed by flow cytometry, this higher accumulation of tracer within the LN from i.d. delivery did not translate into superior delivery to the general population of LN leukocytes for any tracers except the 30 nm PPS NPs (Fig. 4C). However, delivery to barrier macrophages lining the SCS (SSMs) was significantly higher from i.d. relative to i.v. administration for all tracers 10–500 nm in size (Fig. 4D). The only tracer that did not associate with SSMs more from i.d. than i.v. delivery was the 5 nm dextran, perhaps due to limited retention proximal to SSMs because this size is well below the threshold of size-restriction by the SCS.^52–54^ While association with SSMs was also elevated for the 30 nm PPS NP i.d. as compared to i.v., it was the only tracer that was not restricted from overall LN cell delivery to the same extent as the others. This may be explained by the influence of the PEG corona on these PPS NPs, which has been shown to influence transport across lymphatic endothelial cells in a manner dependent upon PEG conformation and grafting density.^55^ PEG influences aside, trends in the majority of tracers exhibited superior LN accumulation from i.d. relative to i.v. administration that resulted in elevated barrier cell delivery but not elevated delivery to LN cells in general, which implicated SCS barrier influences on access to cells within the LN parenchyma from i.d. administration. To probe this further, confocal microscopy was performed on tissue slices from LNs of animals receiving i.v. and i.d. administered 30 nm PPS NP with unique fluorophore labels. NPs administered i.v. were observed in clusters within the central regions of the LN, whereas NPs administered i.d. were predominantly restricted to the peripheral regions of the LN sinus, confirming the effect of administration route on mechanism of entry to the LN (Fig. 4E).

The implications of this on delivery to specific immune cell subsets within the LN were investigated further by normalizing tracer delivery to individual leukocyte populations, measured by flow cytometry, to total LN accumulation of tracer, measured in bulk tissue homogenate. In these calculations, tracer association with cells was normalized to either the percent of injected dose or to the raw mass of tracer accumulated within the LN. To avoid artificially elevating values from i.v. administration simply because of its lesser LN selectivity, normalization to raw mass of tracer was used for further analysis (Fig. S15†). These analyses were performed to assess whether i.v. administered tracers were more efficiently delivered to LN parenchyma-resident cell subsets than i.d. administered tracers as a result of their more direct access to the LN parenchyma through the blood supply. Thus, administration method influences on efficiency of delivery to parenchyma-resident T cells, B cells, and cDCs was assessed and compared to that of barrier sinus lining SSMs.

**Fig. 5:**
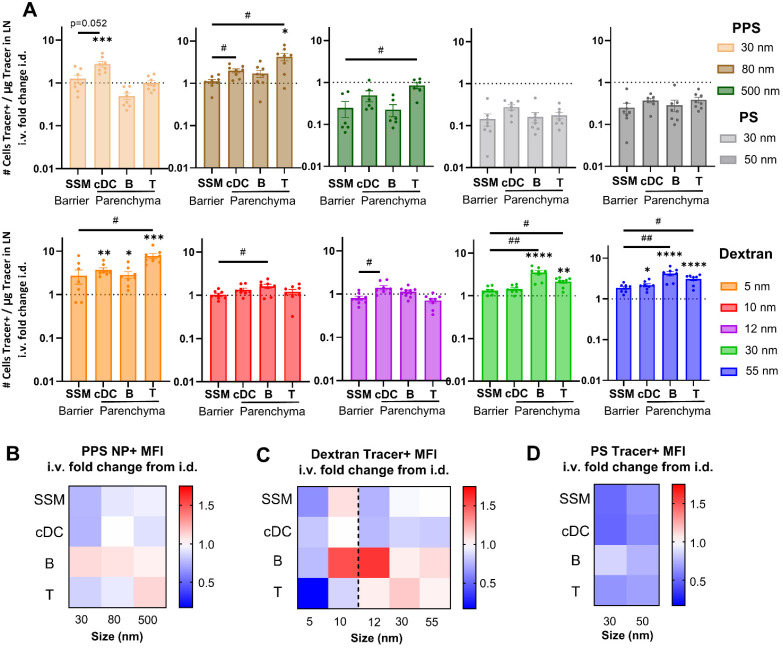
Quantity of tracer association with LN leukocyte subtypes differs between i.v. relative to i.d. routes of administration. (A) Number of cells associated with tracer per microgram of tracer delivered to the lymph node from i.v. administration fold change relative to i.d. administration. *, **, ***, **** indicate significant difference in delivery between i.v. and i.d. administration (*p* < 0.05, *p* < 0.01, *p* < 0.001, *p* < 0.0001, respectively), whereas # and ## indicate significant difference between cell types (*p* < 0.05, *p* < 0.01, respectively) by one-way ANOVA. Heat map of (B) dextran tracer+ MFI, (C) PPS NP+ MFI, and (D) polystyrene tracer+ MFI in major leukocyte subsets from i.v. relative to i.d. administration. Dashed line in (C) represents the size cut-off of the subcapsular sinus and conduit entry in the LN parenchyma. *n* = 5 mice per group and results are representative of two independent experiments.

Relative to i.d. administration, i.v. administration increased association with T and B cells for the 5 nm, 30 nm, and 55 nm dextran tracers, as well as to cDCs for the 5 nm and 55 nm sizes (Fig. 5A). Similarly, i.v. administered 30 nm PPS NPs associated more with cDCs and i.v. administered 80 nm PPS NPs associated more with T cells (Fig. 5A). Even though more overall tracer associates with SSM by i.d. delivery, the same relative amount of the tracer accumulating within the LN associates with the SSM compared to i.v. delivery (Fig. 5A). Therefore, while i.v. and i.d. delivery have the same efficiency in delivering to LN barrier cells, i.v. delivery is much more efficient at accessing leukocytes within the LN parenchyma for the tested tracers. Furthermore, for all dextran and PPS NP tracers, the efficiency of delivery for i.v. compared to i.d. administration was greater in at least one parenchyma-resident cell type relative to barrier SSMs (Fig. 5A). I.v. administered dextran and PPS NP tracers were therefore consistently more efficient than i.d. administered tracers at associating with LN parenchyma-resident cells than barrier cells. Even in the case of the 500 nm PPS NP, which trended toward lower efficiency of association with all cell types from i.v. relative to i.d. administration, this tracer was more efficiently associated with T cells than barrier SSMs from i.v. administration than it was from i.d. administration (Fig. 5A). Deviating from the trends in the dextran and PPS NP tracers, i.v. administered polystyrene tracers displayed no elevated efficiency of delivery to parenchyma-resident cells, and overall less efficient delivery than from i.d. administration (Fig. 5A), in agreement with their overall poor i.v. LN accumulation. As a whole, these analyses into efficiency of tracer delivery to leukocyte subsets suggests that i.v. administration increases tracer delivery to the LN parenchyma, enhancing access to cells in the cortex and paracortex relative to the LN sinus.

To corroborate this finding, quantities of tracer delivered per cell from i.v. and i.d. administration were assessed on the basis of tracer positive MFI. Delivery of the 30, 80, and 500 nm PPS NPs to B cells was higher from i.v. relative to i.d. administration, an effect also observed for the 500 nm PPS NP in T cell delivery (Fig. 5B). No such influence was observed in cDCs and macrophages, typically located in the more sinus-proximal regions of the LN and more accessible from i.d. administration.^56^ For the 5 nm dextran, which is permitted free access to LN conduits when i.d. administered, MFI was lower from i.v. relative to i.d. administration across all cell types (Fig. 5C). The 10 nm dextran, also permitted LN conduit access, showed elevated MFI in B cells and SSMs from i.v. relative to i.d. administration (Fig. 5C). However, in agreement with the PPS NP results, for dextran tracers above the threshold for conduit entrance, i.v. administration enhanced MFI of 12 nm, 30 nm, and 55 nm tracer+ T cells and B cells relative to the MFI of these cell types from i.d. administration (Fig. 5C). No benefits to parenchyma-resident cell delivery were observed in the 30 and 50 nm polystyrene tracers, despite these sizes also being above the conduit threshold, likely because of their poor LN accumulation from i.v. administration, as discussed previously. Results for the 500 nm polystyrene tracer were excluded from these analyses given the nearly negligible quantities of this tracer reaching the LN from i.d. administration at the 4 h time point (Fig. S7†). Overall, these results highlight that trends in tracer quantities delivered to leukocyte subsets i.v. relative to i.d. bias toward increased delivery to cell types located in the lymph node parenchyma and decreased delivery to cell types closer to the SCS barrier of the LN. This finding is consistent with the hypothesis that i.v. delivery can increase access to LN lymphocytes directly through the blood supply that are more difficult to access by lymphatic-mediated delivery, with the stipulation that the material has properties favorable for prolonged circulation after i.v. administration.

### 3.4. Delivery of i.v. administered nanomaterials to LN-resident leukocytes is size-dependent and material-independent when normalized to quantity reaching the LN

**Fig. 6:**
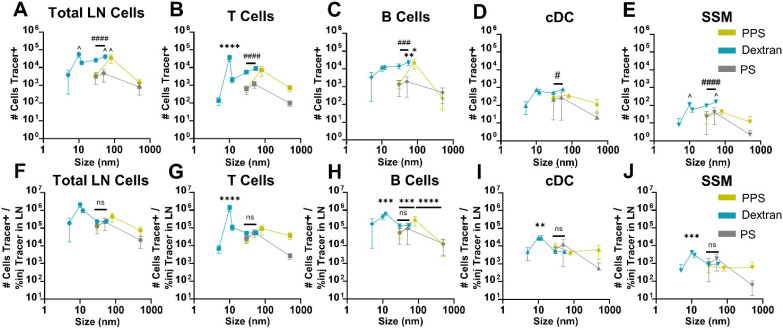
Hydrodynamic size effects of i.v. administered PPS NP, dextran, and polystyrene tracer association with leukocytes within LNs. Number of (A) total cells, (B) T cells (CD3^+^B220^−^), (C) B cells (B220^+^CD3^−^CD11b^−^), (D) conventional dendritic cells [cDC] (CD11c^+^CD3^−^B220^−^), and (E) subcapsular sinus macrophages [SSM] (CD3^−^B220^−^CD11b^+^CD169^+^F4/80^−^CD11c^LO^) in the lymph node associated with tracer 4 h post intravenous administration as a function of tracer hydrodynamic size. (F–J) Data in (A–E) normalized to quantity of tracer reaching the lymph node (% of injected amount) measured in tissue homogenate. *, **, ***, and **** indicate significant difference between tracer sizes by two way ANOVA. ^ indicates significant difference from all other tracer sizes except those also marked with ^ by two way ANOVA. #, ###, and #### indicate significant difference between materials for dextran and both polystyrene and PPS NP by two way ANOVA. *n* = 5 mice per group and results are representative of two independent experiments.

Considering a substantial fraction of current clinical immunotherapies are administered i.v., the size-dependence of nanomaterial delivery to immune cells within the LN resulting from i.v. injection was further investigated. With respect to total cell delivery, the greatest number of cells were accessed by the 10 nm dextran, 55 nm dextran, and 80 nm PPS NP, which outperformed all other sizes, but did not significantly differ from each other (Fig. 6A). This outcome is interesting given the stark contrast in the amount of the 10 nm dextran accumulating in the LN relative to the 55 nm dextran and 80 nm PPS NPs (Fig. 2E). These results highlight the competing influences of size on the quantity of tracer reaching the LN and the access of tracer afforded to cells within the LN structure.

Given the compartmentalized nature of the distribution of leukocyte subsets within the LN, these analyses were next carried out more granularly by cell subtype. With respect to access to the T cell compartment, the 10 nm dextran was superior to all other tracer sizes, even despite its lower total LN accumulation (Fig. 6B). However, in the B cell compartment, total LN accumulation was more influential, as the 55 nm dextran and 80 nm PPS NP accessed B cells to a greater extent than all other sizes (Fig. 6C), in line with size trends of total LN delivery. The influence of size was less pronounced on delivery to cDCs, which were accessed to greater extents by the 10 nm dextran relative to the smaller 5 nm dextran, but no size dependent trends at larger sizes were notable (Fig. 6D), whereas in the barrier macrophages (SSMs), delivery was greatest for the 10 nm and 55 nm dextrans (Fig. 6E).

We sought to decouple the influences of total LN accumulation and movement within the LN structure to hone in on the size-dependence of i.v. delivery to leukocytes by normalizing leukocyte association with tracer (assessed through flow cytometry, Fig. 6A) to the percent of injected tracer quantity reaching the tissue (measured using bulk tissue homogenate fluorescence, Fig. 2E). Interestingly, when adjusted for quantity of tracer in the LN, access to leukocytes for i.v. administered dextran and polystyrene tracers and PPS NPs of similar size converged (Fig. 6F). This implicates hydrodynamic size as a more influential property than material on the delivery to LN leukocytes per unit quantity delivered to the tissue from i.v. administration. This normalized data provides insights into the “efficiency” of delivery to cells for materials of varied size, of interest from a mechanistic perspective to understand size influences on cell access independent of total delivery. Efficiency of delivery to LN cells increased between the sizes of 5 to 10 nm, where it peaked, then decreased with increasing nanomaterial size from 10 to 500 nm (Fig. 6F). This trend was also generally conserved in T cells, B cells, cDCs, and SSMs (Fig. 6G–J), however, in the case of B cell delivery, efficiency reached an additional local maximum at 80 nm in size (Fig. 6H). This outcome appeared to be tied to the size-dependence of tracer access to cells within the LN *in vivo*; when tracers were incubated with cells isolated from murine LNs for 4 h *in vitro*, tracer association differed substantially (Fig. S16†). Following *in vitro* incubation, efficiency of cell association was clearly material-dependent, as the PPS NPs and polystyrene tracers were more efficiently taken up than the dextran tracers in the 30–80 nm size range, with no convergence of size trends being evident between materials, contrasting from the outcome from i.v. injection *in vivo*. Thus, the material-independent size trends of access to cells from i.v. administration appear dependent upon the access of tracer to cells within the LN, which is predominantly regulated by hydrodynamic size.

## 4. Discussion

Enhancing drug delivery to immune cell targets within the LN has the potential to improve therapeutic outcomes in a variety of applications, including cancer immunotherapy. While therapeutic delivery to the LN *via* locoregional methods of administration is well described, properties favorable for accumulation of systemically administered materials within the LN are less understood, as is the understanding of how these properties vary compared to those favorable from locoregional injection. This is important given that many current immunotherapy treatments are administered i.v., and i.v. delivery to the LN may be a particularly potent approach to broadly drug systemic leukocytes or for treatment of LN tumors, either primary or metastatic. Within the LN, delivery to leukocytes of interest, including B lymphocytes, T lymphocytes, conventional dendritic cells, and subcapsular sinus macrophages, was evaluated because these immune cells, as important cell populations for modulating the adaptive immune response, are the most common targets for immunotherapies.

Nanomaterials have been shown to improve bioavailability of systemically administered treatments because of their size,^10^ and size also plays a significant role in lymphatic delivery,^17,30^ as well as movement throughout the LN.^37^ The size ranges of the tracers in these studies were selected because they enable favorable lymphatic accumulation from locoregional administration^20,27,41,57^ and favorable circulation time in blood.^46^ Macromolecule dextran tracer sizes were additionally selected to probe the size range of lymphatic-mediated conduit exclusion from the SCS, however, could not be consistently synthesized in the range of hundreds of nanometers in hydrodynamic size like the larger PPS and PS particle tracers. In addition to size, other aspects of nanomaterial physical properties are also known to influence their biodistribution, by modulating blood circulation time, clearance by systemic organs, and uptake by cells.^58^ Thus, utilizing a panel of tracers with varied physical properties was valuable in these studies designed to interrogate size- and material-dependent trends on LN delivery, and notably convergence of size trends between materials indicates LN delivery is less tied to specific materials. While the tracer size was the primary variable considered in these studies, additional differences between these tracers should also be noted. Dextran is a relatively flexible, branched macromolecule commonly used *in vivo* for transport studies^52,59,60^ and as a coating for nanoparticles to increase their biocompatibility.^61–63^ By contrast, the polystyrene tracers used in these studies are more rigid^20^ and hydrophobic,^64^ characteristics reflected in their poorer circulation time and generally low accumulation within the LN *in vivo*. The PPS NPs used in these studies are a softer nanoparticle with a PEGylated corona affording them long circulation time. The dextran tracer and PPS NPs showed elevated LN accumulation in the 30–80 nm size range, while this trend was not observed in the polystyrene particles. Given that both dextran and PPS NPs are generally considered hydrophilic (at their surface at least) and longer circulating than polystyrene, agreement of size trends between these materials may indicate this trend would be conserved in other similarly biocompatible materials. The polystyrene tracer data serves as an important counterpoint that while size is clearly influential on LN delivery, it is not the only material property of importance, and size benefits cannot as readily be leveraged for materials poorly suited for i.v. circulation and lymphatic drainage.

We note that when considering the LN accumulation of different tracer materials in these studies 4 h post administration, the trends of size dependence are more important than the comparative total amounts reaching the LN. While the long circulation time of the PPS NPs was shown to be beneficial for i.v. LN accumulation over time, a substantial portion of the injected dose was still in the blood at the majority of time points studied and therefore had not distributed to tissues. Thus, the total quantity of PPS NP accumulated within the LN within 4 h was limited in this regard, while much less influential in the other tracer systems that had already predominantly been cleared from the blood within 1–4 h. While varied extents of total delivery between these tracers may have been measured at different time points of analysis, the focus of this study was to delineate size-dependent trends across multiple materials rather than optimal accumulation of one particular material. Therefore, these studies represent size-dependent trends but do not necessarily imply superiority of LN accumulation for one i.v. administered material over another.

The majority of the i.v. administered dextran and polystyrene tracers were cleared from the blood within 4 h, yet for some tracers, LN accumulation was found to increase from 4 to 24 h post-injection. This delayed LN accumulation may have resulted from tracer extravasation from the blood in peripheral tissues and subsequent draining to the LNs through lymphatics over longer time frames,^16,65^ or being trafficked there by cells.^41^ Considering the role of lymphatic-mediated entry in these suggested later-stage mechanisms of i.v. administered LN accumulation, the benefits to LN parenchyma-resident cell type delivery observed from i.v. relative to i.d. administration 4 h post-injection may diminish at later time points. Nonetheless, direct LN parenchyma access remains an important difference in the mechanism of entry to the LN from i.v. administration relative to local administration methods that can be expected to influence quality of cell access between these administration methods independent of time.

In studies comparing administration routes, i.d. delivery achieved superior total LN accumulation, but also greater association with barrier macrophages, whereas i.v. delivery biased association with lymphocytes and increased the quantity of tracer delivered per lymphocyte, as measured by tracer signal MFI, when compared to i.d. delivery. This outcome may be explained by LN structural studies, which have shown that the highest densities of capillaries and HEVs in the LN are in the interfollicular regions of the cortex and the peripheral regions of the paracortex.^66^ Therefore, therapeutic delivery through the blood is likely to improve delivery to (para)cortex- and parenchyma-resident cells rather than sinus-lining barrier cells.

These studies were designed to assess differences in cell association from locoregional compared to systemic administration from a mechanistic standpoint rather than comparing total LN accumulation. To accomplish this, accumulation in only one LN was considered in these analyses. In the case of i.d. administration, this was the primary draining LN that receives the bulk of the tracer, whereas from i.v. administration, this was just one representative LN. Therefore, while total LN accumulation and total tracer delivery to LN leukocytes throughout the body could not be compared between administration methods, these studies provided insight into the extent to which administration route could be utilized to alter leukocyte access within the LN. The transport barrier phenomena observed in these studies in mice can be expected to also apply to humans because the sinus structure of the LN is largely conserved between species.^67^ However, in humans, LNs are larger in size and therefore total tissue volume,^68^ and also contain more complex sinus structures including transverse sinuses that result in greater sub-compartmentalization of LN lobules.^66,67^ Because of the greater size and volume of LNs in humans, transport barriers that limit access from the LN sinuses to the parenchyma may be even more significant in humans than in mice, though this warrants future studies. Ultimately, the different routes of administration have respective advantages and limitations. While i.v. administration biases delivery towards leukocytes within the LN parenchyma, the systemic exposure of the immunotherapy may limit the tolerable dose. I.d. administration, however, localizes the therapeutic to the region surrounding the injection site, but the delivery is predominantly to the SSMs within the LN. In particular, the target cells of interest and potential side effects are some of the many considerations when designing immunotherapies.

On the basis of “efficiency” of delivery to cells adjusted for amount reaching the tissue, material-dependence of cell association became insignificant and tracers of all materials converged to size-dependent trends. While efficiency of delivery to cDCs and SSMs followed very similar size-dependent trends, this analysis revealed differences in design principles for efficient i.v. delivery to LN resident T cells and B cells. Delivery to T cells was maximal with respect to extent and efficiency at the 10 nm size range, even when compared to larger material sizes accessing the LN in greater quantities, whereas efficiency of delivery to B cells peaked at 12 nm with a secondary, but lesser, local maximum at 80 nm. This result indicates that even for materials administered into the blood supply, transport through the LN structure of larger solutes may be limited, and materials small enough to freely move through LN structural features such as the conduit system^52–54^ achieve substantially more efficient access to T cells. By contrast, extent of delivery to B cells was greatest for 55–80 nm materials that accumulated in the LN in the highest quantities, but nonetheless, efficiency of B cell delivery was higher in the range of 10–12 nm. Thus, even under apparently less restriction of access by LN structure, delivery to B cells was still more efficient for materials smaller in size than those more optimal for total tissue accumulation.

## 5. Conclusions

This study provides new insights into the ways nanomaterial access to immune cells in LNs changes from i.v. relative to local i.d. administration and characterizes nanomaterial delivery to the LN from systemic administration. While size and material profiles of nanomaterial delivery to the LN are similar from systemic and local administration routes, when considering delivery to immune cell subsets, i.v. administration improves tracer quantities delivered to lymphocytes in the LN parenchyma for tracer sizes generally restricted by the SCS when administered locoregionally. Size and material characteristics of nanomaterials affect extents of delivery to LN-localized cells from i.v. administration, but when adjusted for quantity in the LN, cell access of i.v. administered materials is strongly regulated by size. These findings reveal properties of i.v. administered nanoformulations favorable for enhancing LN accumulation and lymphocyte delivery to improve efficacy of systemically administered immunotherapies.

## Author contributions

P. A. A.: conceptualization, formal analysis, investigation, writing – original draft, writing – review and editing, visualization. A. J. H.: investigation, writing – review and editing. A. R. B.: investigation, writing – review and editing. Y. A.: investigation, writing – review and editing. S. N. T.: conceptualization, writing – original draft, writing – review and editing, supervision, project administration, funding acquisition.

## Data availability

Data pertaining to tissue accumulation and cellular association of fluorescent tracers are available upon request from the authors.

## Conflicts of interest

Authors declare no conflicts of interest in this publication.

## Supplementary Material

BM-012-D4BM00910J-s001
