# Supplementary material for: Different leukocyte subsets are targeted by systemic and locoregional administration despite conserved nanomaterial characteristics optimal for lymph node delivery
Source: Biomater Sci. 2024 Sep 18;12(21):5582–97. doi: 10.1039/d4bm00910j (PMC11422756; doi:10.1039/d4bm00910j)
Supplement: BM-012-D4BM00910J-s001 [file BM-012-D4BM00910J-s001.pdf]

Supplementary Information (SI) for Biomaterials Science.
This journal is © The Royal Society of Chemistry 2024

A

	Size (nm)	Tracer concentration injected (mg/mL)	Fluorescent dye Excitation/Emission (nm)
**PPS NP**	30	22.5	Alexa Fluor 647 (645/665)
	80	22.5	Alexa Fluor 647 (645/665)
	500	45	Alexa Fluor 647 (645/665)
**Dextran**	5	0.25	Alexa Fluor 647 (645/665)
	10	2.5	Alexa Fluor 647 (645/665)
	12	2.5	Texas Red (595/615)
	30	2.5	Alexa Fluor 700 (700/720)
	55	2.5	TRITC (555/575)
**PS**	30	2.5	Crimson (625/645)
	50	2.5	Orange (540/560)
	500	2.5	Red (580/605)

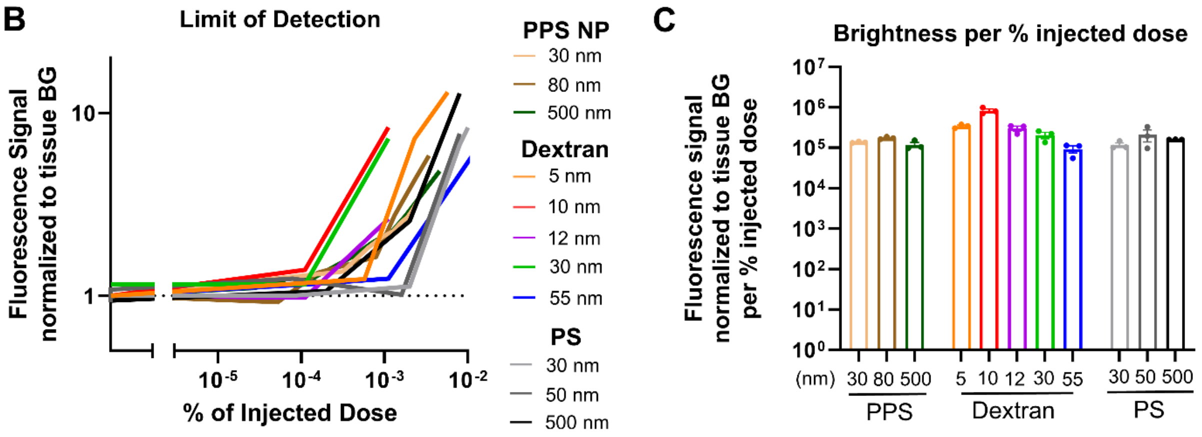

Fig. S1: Selection of injected tracer quantities for tissue homogenate biodistribution experiments on the basis of comparable fluorescence detection limits. A) Overview of tracer quantities administered in tissue homogenate biodistribution experiments and fluorophore labels on each tracer. B) Percentage of injected dose at which tracer fluorescence signal approaches background fluorescence, designated the limit of detection. C) Detectability of each tracer relative to respective fluorophore background signal on the basis of percent of injected dose.

A

	Size (nm)	Tracer concentration injected (mg/mL)	Fluorescent dye Excitation/Emission (nm)
**PPS NP**	30	40	Texas Red (595/615)
	80	40	Alexa Fluor 488 (490/525)
	500	50	Alexa Fluor 647 (645/665)
**Dextran**	5	0.35	TRITC (555/575)
	10	3.5	Alexa Fluor 647 (645/665)
	12	3.5	Texas Red (595/615)
	30	3.5	Alexa Fluor 700 (700/720)
	55	3.5	TRITC (555/575)
**PS**	30	3.5	Yellow-green (505/515)
	50	3.5	Dark Red (660/680)
	500	3.5	Red (580/605)

B

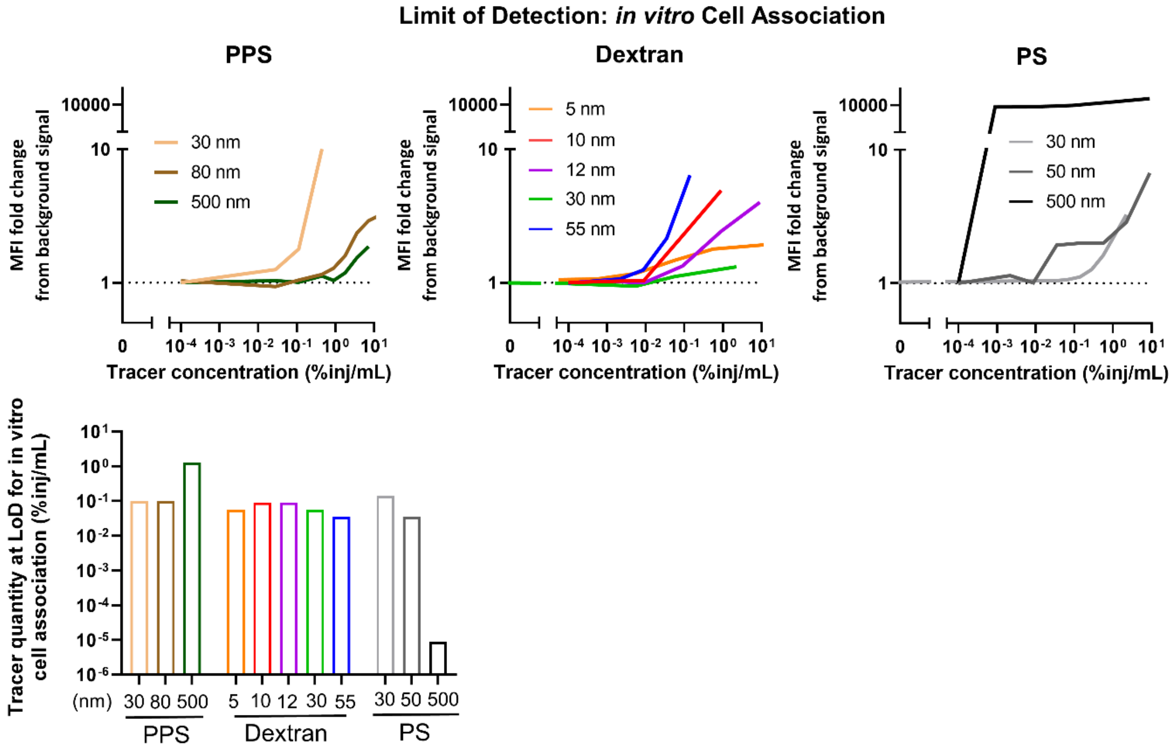

Fig. S2: Selection of injected tracer quantities for flow cytometry cellular distribution experiments based on comparable detection limits of cell association. A) Overview of tracer quantities administered in flow cytometry experiments and fluorophore labels on each tracer. B) Percent of injected dose at which limit of detection is reached for tracer association with splenocytes *in vitro*, based on median fluorescence intensity (MFI) of tracer signal becoming

indistinguishable from background. C) Tracer concentration at which median fluorescence signal intensity approaches background for splenocytes incubated with tracer solutions *in vitro* when measured by flow cytometry.

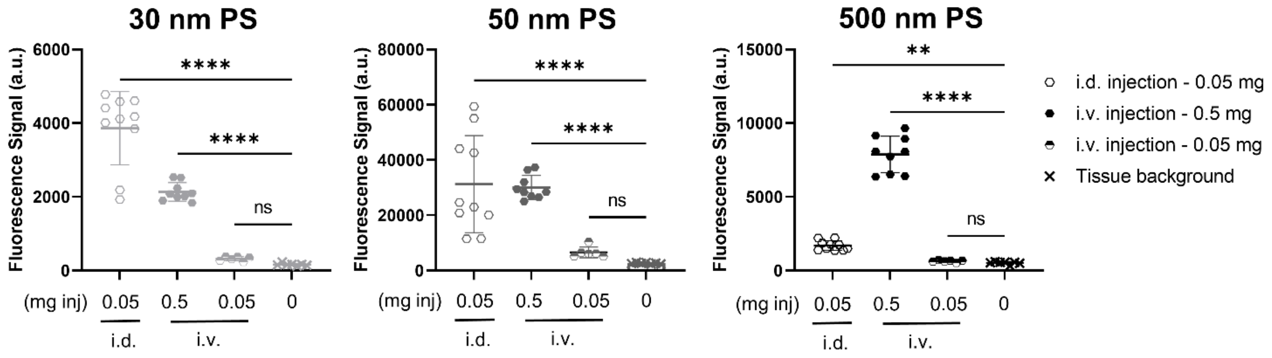

Fig. S3. Dose dependence of fluorescence detection within the LN for i.v. administered polystyrene tracers and limit of detection relative to LN tissue background. Tracer was administered at varied dosages to identify injected amount necessary for detection in the LN from i.v. and i.d. administration. ** and **** indicate significant difference by one-way ANOVA (p<0.01 and p<0.0001, respectively), ns = not significant. I.d. dosage was limited by volume that could be administered into skin, and this dose was not detectable in the LN when i.v. administered, so tracer was administered at tenfold greater volume for i.v. injections. Polystyrene tracer data shown here is representative of dosage-dependence of detection for all tracers.

A

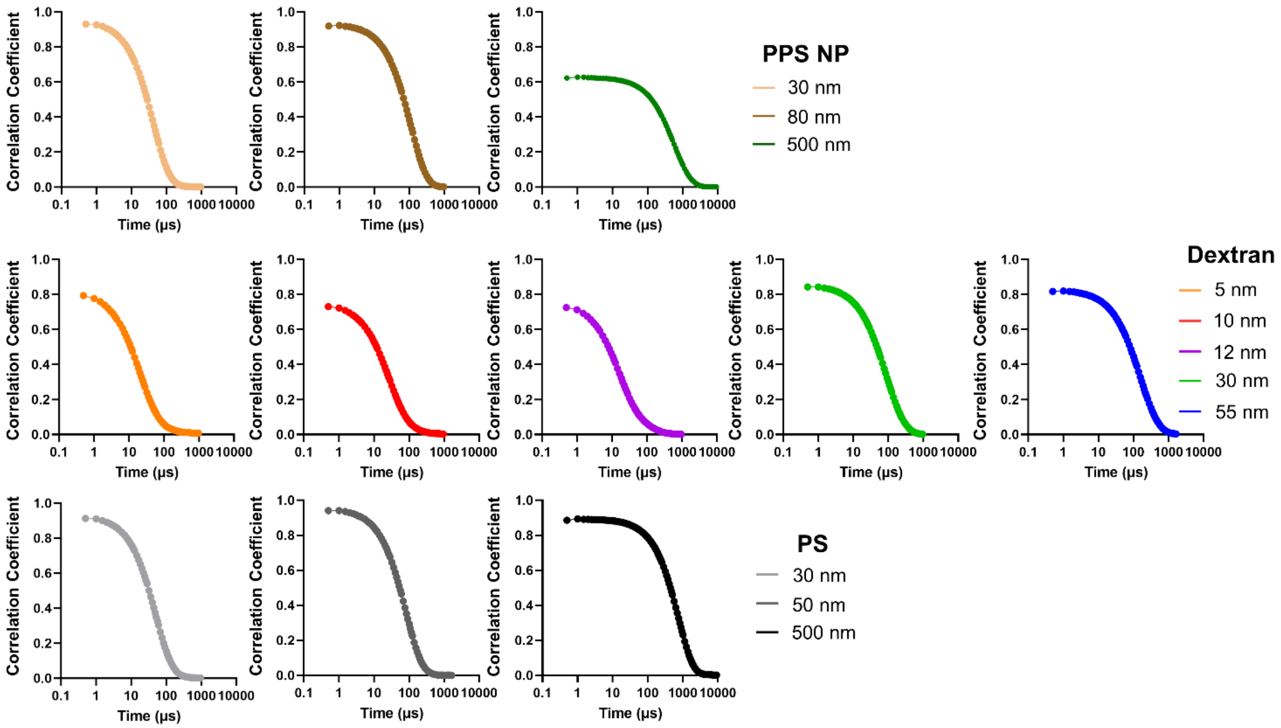

B

	Size (nm)	Polydispersity Index (PDI)
PPS NP	30	0.13
	80	0.07
	500	0.19
Dextran	5	0.345
	10	0.361
	12	0.436
	30	0.274
	55	0.270
PS	30	0.181
	50	0.102
	500	0.083

Fig. S4. Dynamic light scattering measurement details for PPS NP, dextran, and PS tracers. A) Correlogram measurement and B) polydispersity index (PDI) for PPS NP, dextran, and PS tracers.

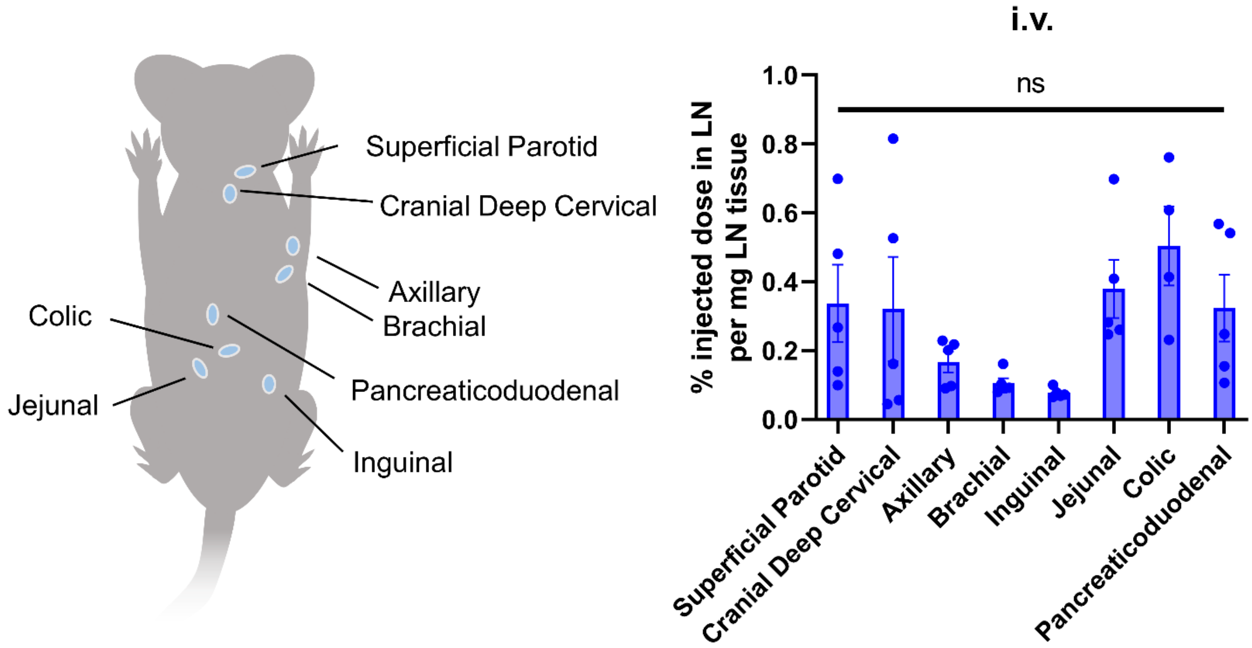

Fig. S5. Intravenously administered tracer accumulation in systemically distributed LNs. Colic, jejunal, and pancreaticoduodenal LNs are found deep within the body cavity near the stomach and intestines, whereas superficial parotid, cranial deep cervical, axillary, brachial, and inguinal LNs drain more peripheral tissues. Intravenously administered tracer accumulates within each LN to a similar extent independent of its position within the body. ns indicates no significant difference between any groups as determined by one-way ANOVA.

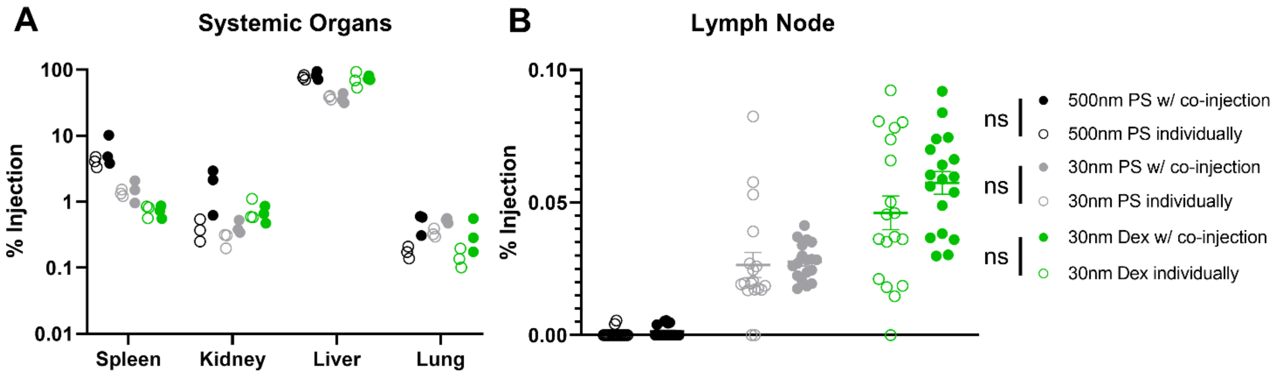

Fig. S6. Characterization of *in vivo* biodistribution for individual fluorescent tracers and mixtures of fluorescent tracers administered intravenously. Tracers co-injected i.v. into the same animal accumulate in systemic organs (A) and lymph nodes (B) to extents that do not significantly differ from tracers separately injected into different animals 4h post-injection. ns = not significant by one-way ANOVA.

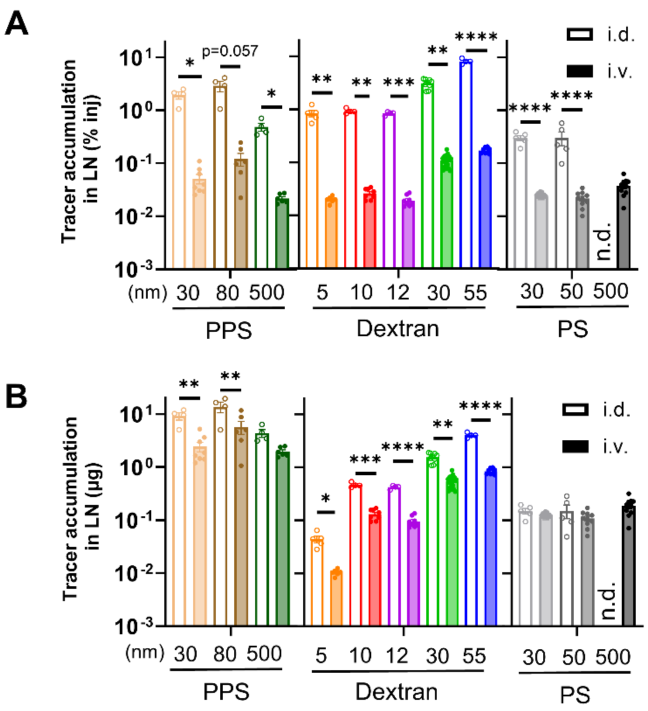

Fig. S7. Comparison of PPS, dextran, and PS tracer accumulation within the LN 4 h post i.v. and i.d. administration. A) Percent of injected dose and B) microgram amount of tracer accumulating in the LN from intravenous as compared to intradermal administration of tracer for PPS, dextran, and polystyrene materials. *, **, ***, **** indicate significant difference between groups by Welch ANOVA.

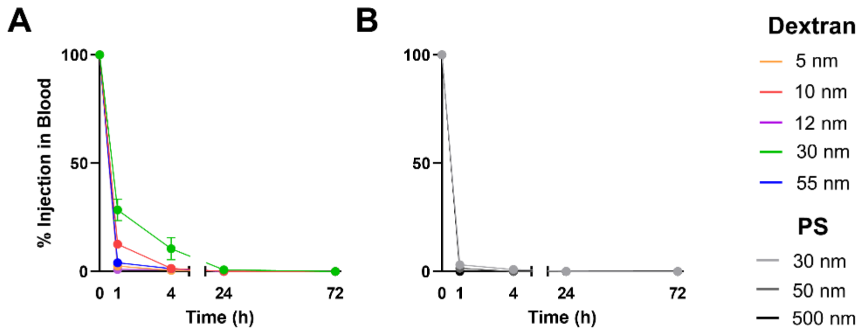

Fig. S8. Blood clearance profile for dextran and polystyrene tracers. Percent of injected tracer remaining in the blood post i.v. injection for A) dextran and B) polystyrene tracers.

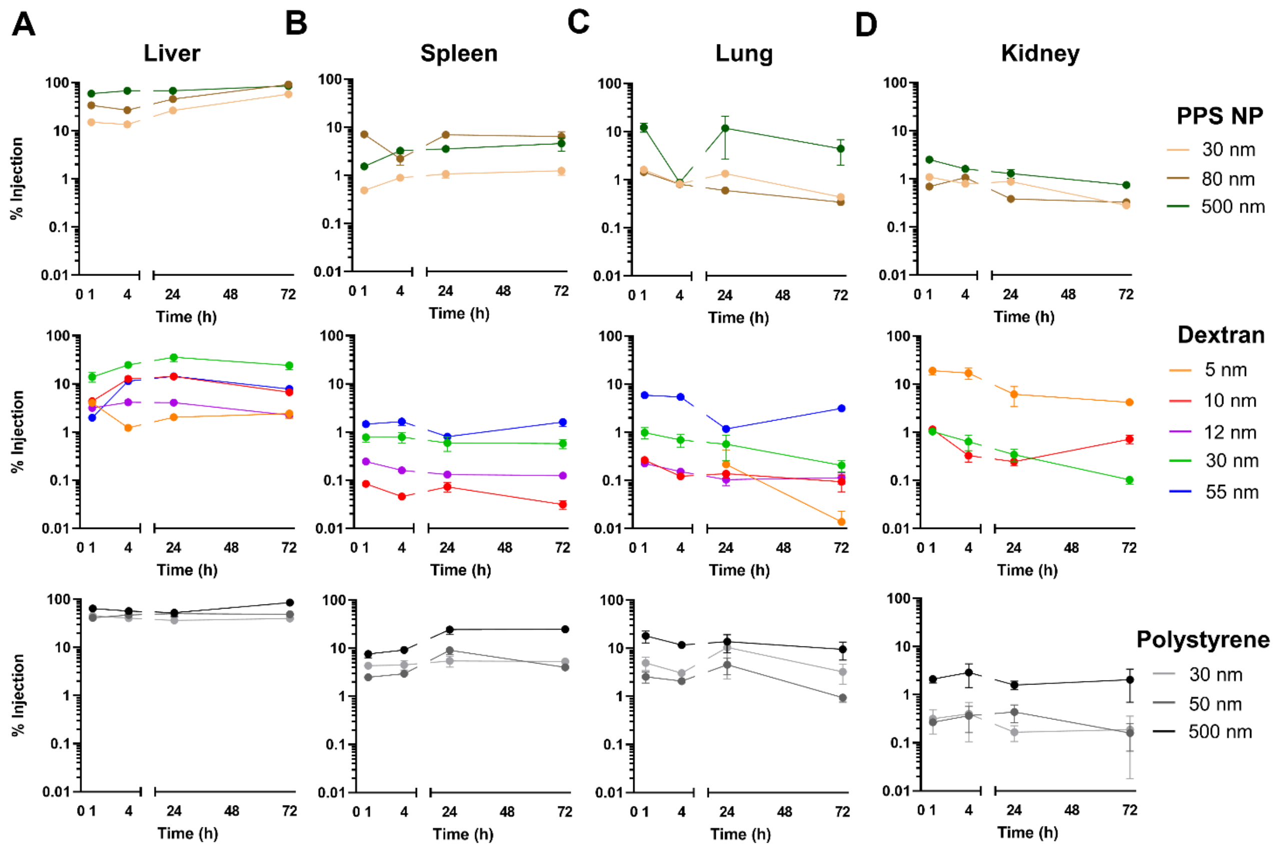

Fig. S9. Biodistribution of dextran, PPS, and polystyrene tracers to systemic organs over 72 h post i.v. administration. Percentage of initial injected quantity of dextran, PPS, and polystyrene tracers detected in the (A) liver, (B) spleen, (C) lungs, and (D) kidneys at 1, 4, 24, and 72h post i.v. injection.

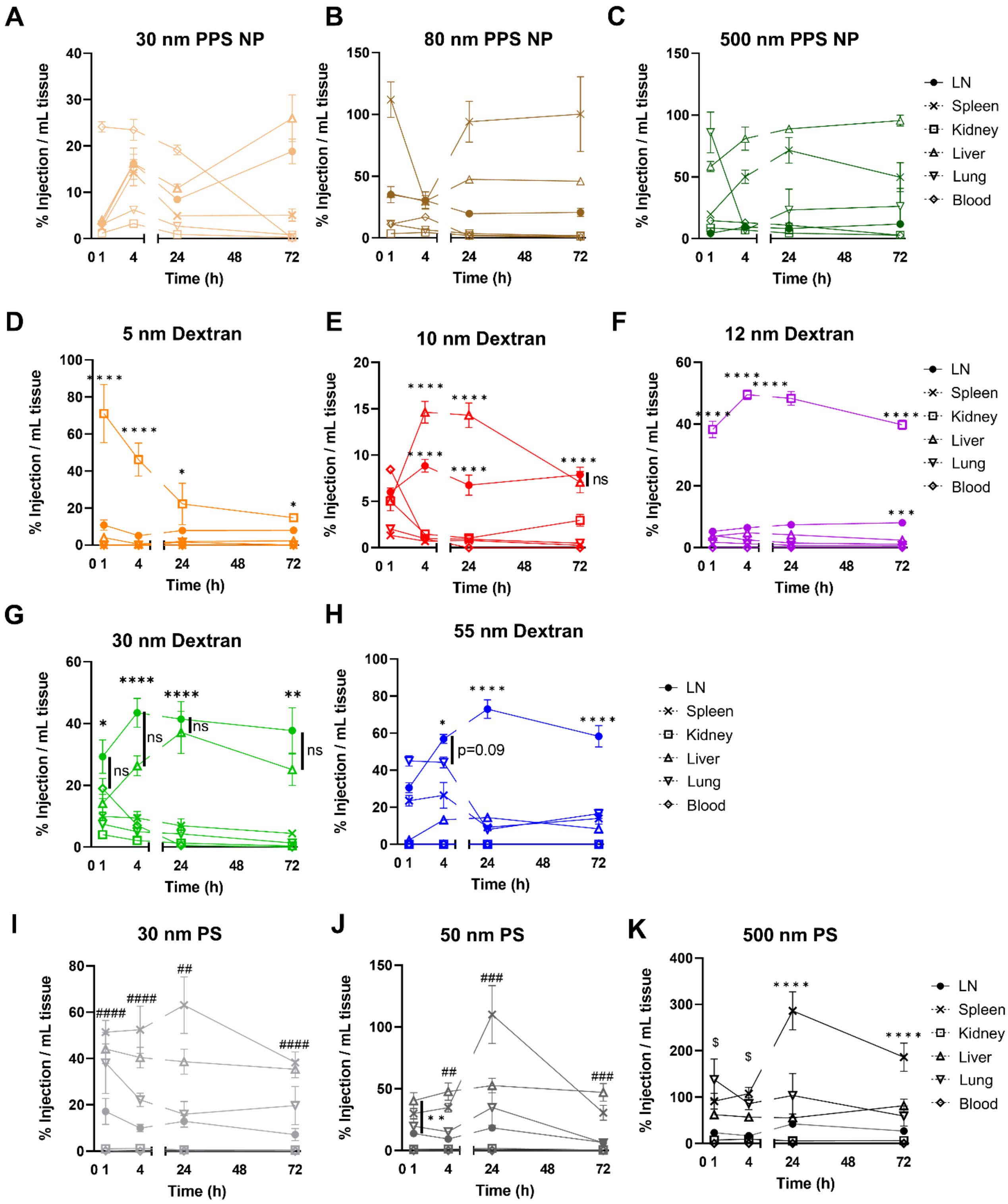

Fig. S10. Percent of injected tracer dose per total tissue volume within LN, spleen, kidneys, liver, lungs, and blood for dextran, PPS, and polystyrene tracers following i.v. administration. Tracer biodistribution of (A) 30 nm PPS, (B) 80 nm PPS, (C) 500 nm PPS, (D) 5 nm dextran, (E) 10 nm dextran, (F) 12 nm dextran, (G) 30 nm dextran, (H) 55 nm dextran, (I) 30 nm polystyrene, (J) 50 nm polystyrene, and (K) 500 nm polystyrene presented as percent of

injected quantity per mL tissue. *, **, ***, **** indicate significant difference between tissues ($p < 0.05$, 0.01, 0.001, 0.0001, respectively) by two way ANOVA. ##, ###, #### indicate significant difference between both spleen and liver compared to lymph node ($p < 0.01$, 0.001, 0.0001, respectively) by two way ANOVA. $ indicates significant difference between both spleen and lung compared to lymph node ($p < 0.05$) by two way ANOVA, ns = not significant.

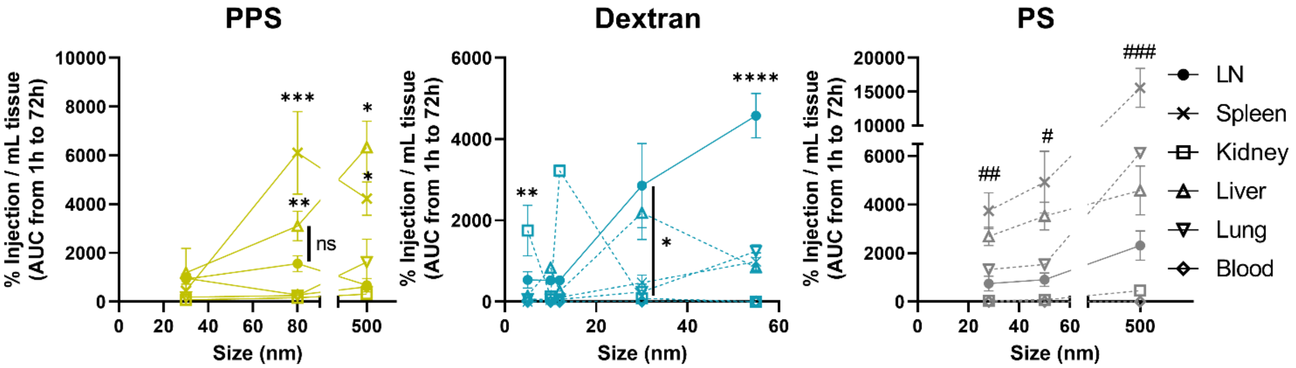

Fig. S11. Total amount of tracer, measured as the area under the curve (AUC) of the percent of injected tracer dose per total tissue volume, delivered to the LN, spleen, kidneys, liver, lungs, and blood presented as a function of size. *, **, *** (p<0.05, 0.01, 0.001, respectively) indicate significant difference from other tissues by Welch ANOVA. #, ##, ### (p<0.05, 0.01, 0.001, respectively) indicate significant difference between spleen and liver from all other tissues.

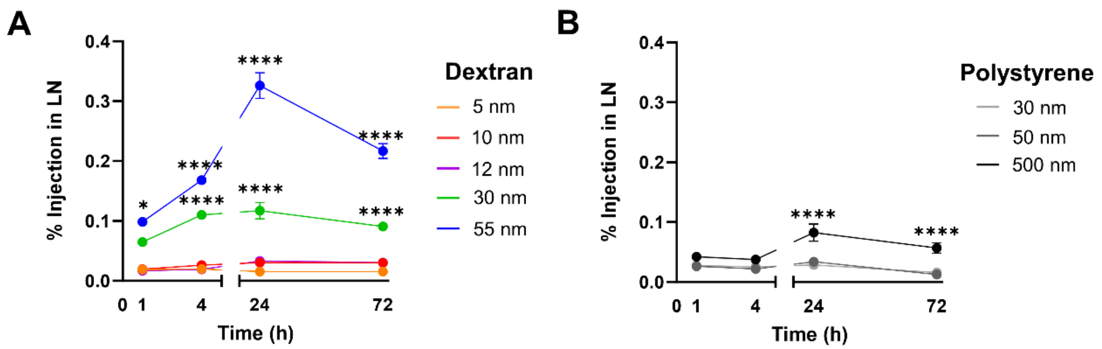

Fig. S12. Dextran and polystyrene tracer accumulation within the LN over 72 h post i.v. administration. Time resolved dextran (A) and polystyrene (B) accumulation within the brachial LN, presented as percentage of injected quantity. * and **** indicate significant difference (p<0.05 and 0.0001, respectively) between designated tracer size and all other tracer sizes at the specified time point.

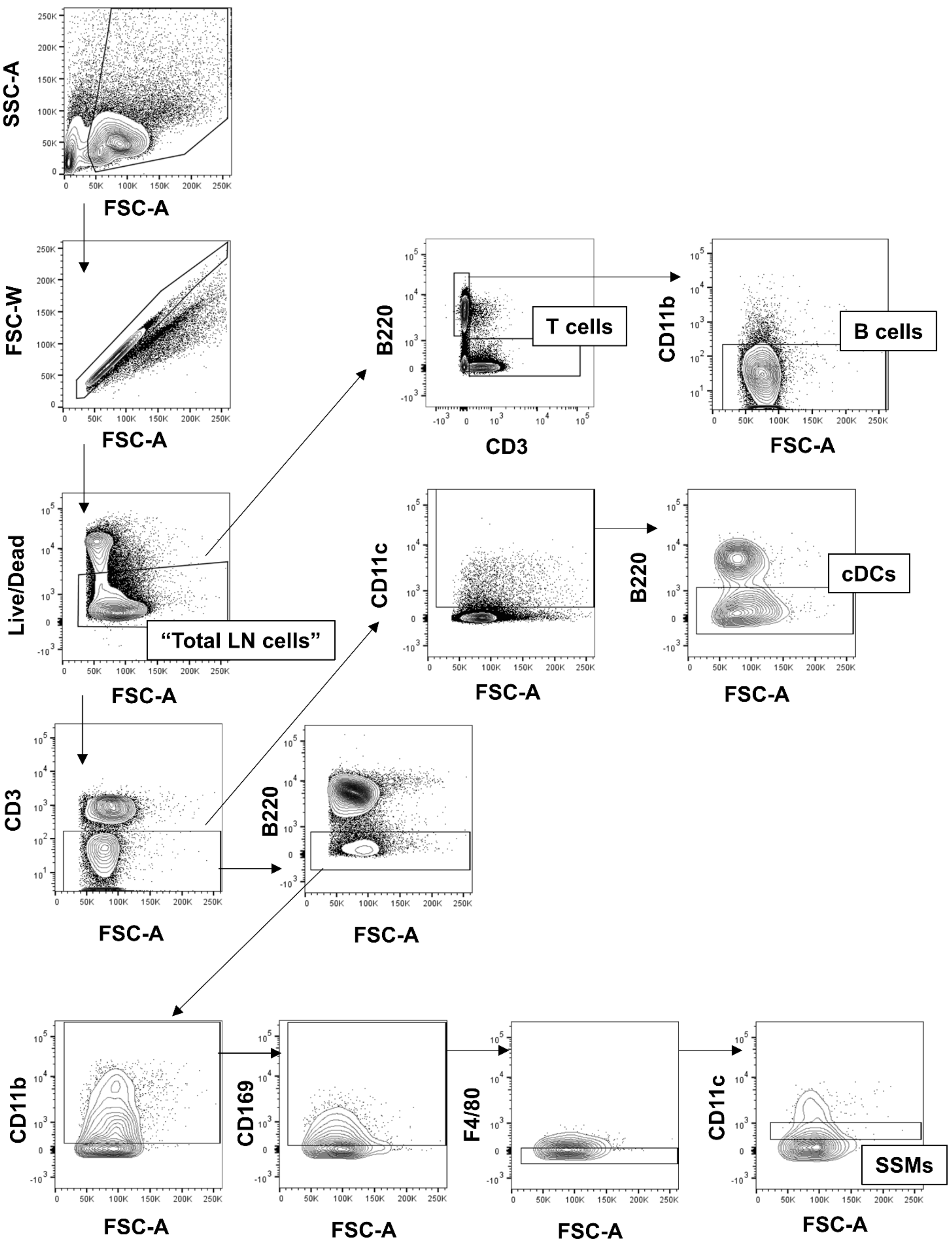
SSC-A
FSC-A
FSC-W
FSC-A
Live/Dead
"Total LN cells"
FSC-A
CD3
FSC-A
B220
CD3
T cells
CD11b
FSC-A
B cells
CD11c
FSC-A
B220
FSC-A
cDCs
B220
FSC-A
CD11b
FSC-A
CD169
FSC-A
F4/80
FSC-A
CD11c
FSC-A
SSMs

Fig. S13. Flow cytometry gating strategy for phenotyping of leukocyte subsets within the LN.

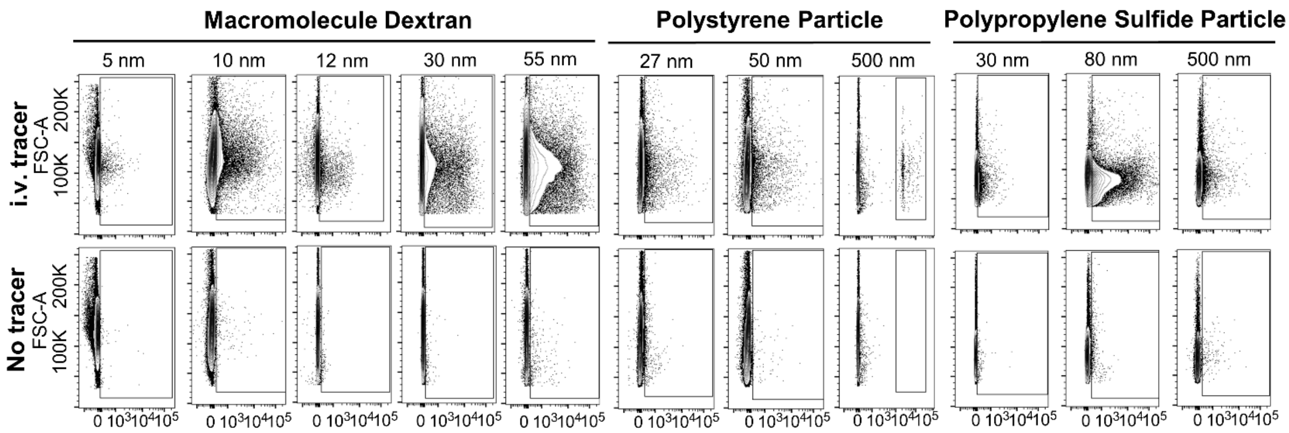

Fig. S14. Representative flow cytometry dot plots displaying fluorescent tracer signal in the brachial LN of mice that received i.v. administered tracer (top) and saline control mice that received no tracer (bottom).

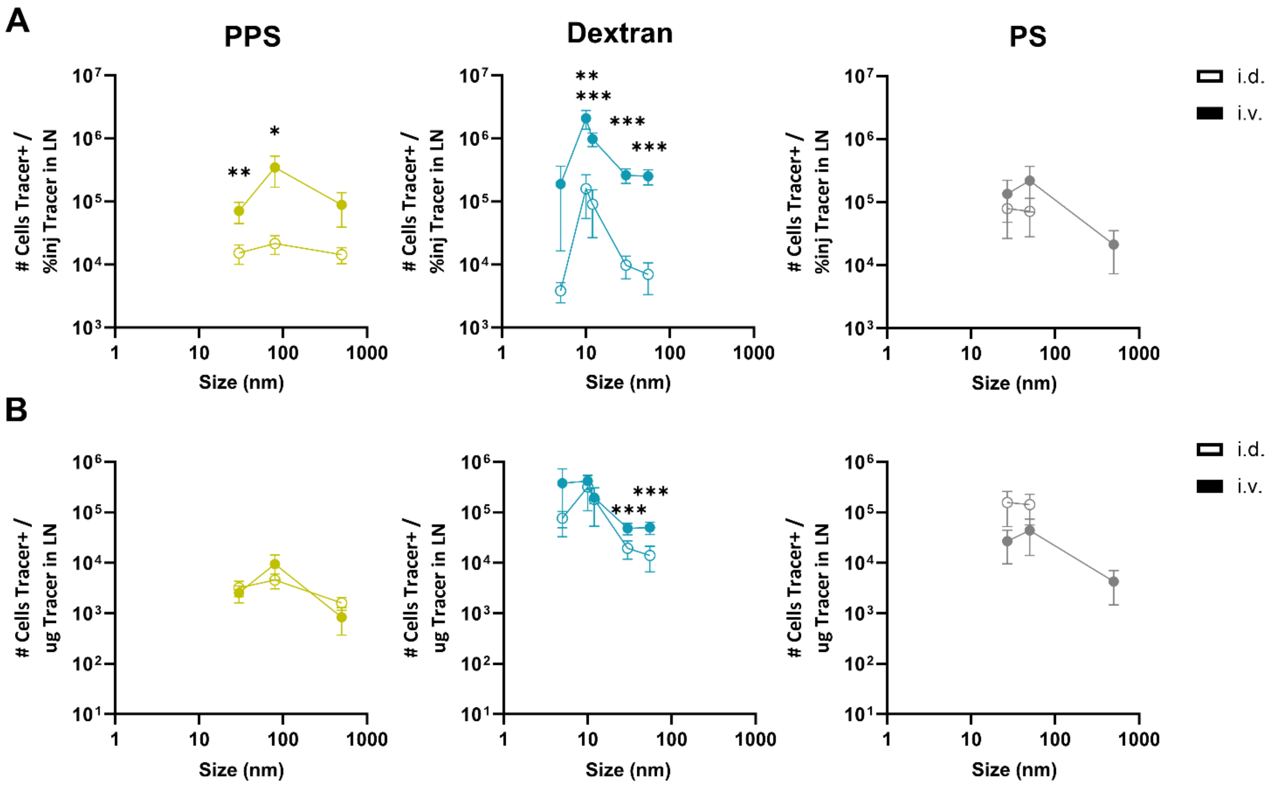

Fig. S15. Tracer delivery efficiency based on cell association normalized by LN accumulation from i.v. compared to i.d. administration. A) Number of tracer positive cells normalized to percent of injected dose accumulated within the LN from i.v. as compared to i.d. administration, presented as a function of tracer size. B) Number of tracer positive cells normalized to tracer mass accumulated within the LN from i.v. as compared to i.d. administration, presented as a function of tracer size. *, **, and *** indicate significant difference between administration routes (p<0.05, 0.01, 0.001, respectively) by Welch ANOVA.

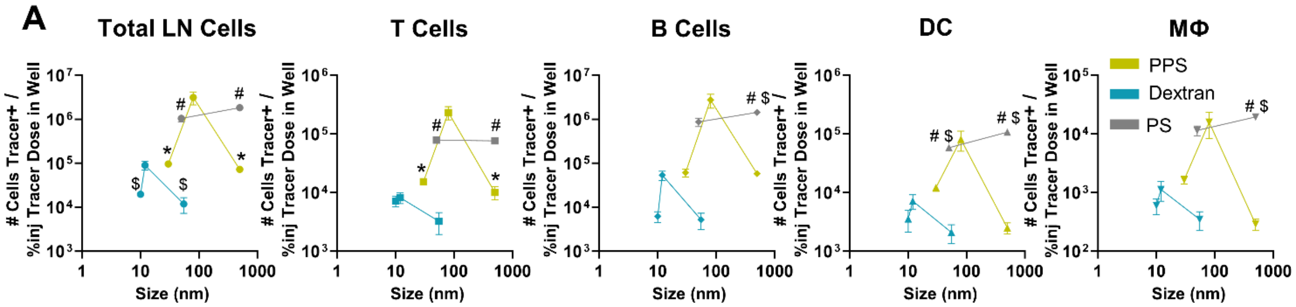

Fig. S16. Size dependence of tracer association with cells after 4 h *in vitro co*-incubation with tracer solution. A) Number of tracer+ splenocytes normalized to quantity of tracer incubated *in vitro*, presented as percent of injected quantity administered *in vivo*. * indicates significant difference from all polystyrene tracers, # indicates significant difference from all dextran tracers, and $ indicates significant difference from 30 and 50 nm PPS NP tracers ($p<0.05$) by Welch ANOVA.
